# Mechanistic interplay between extracellular vesicles and neutrophil extracellular traps: unveiling the “sterile inflammation” cascade in obesity-induced diabetes progression

**DOI:** 10.3389/fimmu.2026.1865003

**Published:** 2026-07-30

**Authors:** He Gao, Xin-xin Luo, Yu Fu, Yi Chen, Shun-yu Ning, Xue Han, Li Wang, Qing-feng Wang

**Affiliations:** 1 Liaoning University of Traditional Chinese Medicine, Shenyang, China; 2 The First Affiliated Hospital of Liaoning University of Traditional Chinese Medicine, Shenyang, China

**Keywords:** extracellular vesicles, insulin resistance, neutrophil extracellular traps, NLRP3 inflammasome, obesity, peptidylarginine deiminase 4, sterile inflammation, type 2 diabetes

## Abstract

Obesity-driven type 2 diabetes mellitus is sustained by chronic low-grade sterile inflammation. The intercellular mechanisms underlying this self-amplifying state remain incompletely defined. Extracellular vesicles and neutrophil extracellular traps have each been independently implicated in metabolic inflammation. Whether they form a coordinated pathological axis in the obesity/type 2 diabetes microenvironment has not been systematically synthesized. This narrative review integrates current mechanistic evidence in three areas: vesicle biogenesis and cargo remodeling in obesity; trap formation in the hyperglycemic and hyperlipidemic milieu; and molecular cross-talk between the two systems. We attend explicitly to the level of clinical evidence supporting each step. Our central argument is the following. Adipocyte- and macrophage-derived vesicles enriched in pro-inflammatory microRNAs activate neutrophils to undergo NETosis. The resulting traps release damage-associated molecules. These molecules stimulate further vesicle secretion. A self-reinforcing positive feedback circuit emerges. It operates through Toll-like receptor signaling, the NLRP3 inflammasome, peptidylarginine deiminase 4, and the cyclic GMP-AMP synthase–stimulator of interferon genes axis. The vesicle pool in obesity is not uniformly pathogenic. Insulin-sensitizing populations also exist. Hepatocyte exosomes from early-onset obese mice are enriched in microRNA-3075 and improve insulin sensitivity. Small vesicles from rosiglitazone-treated adipose tissue macrophages are enriched in microRNA-690 and exert similar effects. Any therapeutic strategy must preserve these protective populations. The pathogenic arm propagates local adipose inflammation systemically. This drives insulin resistance, pancreatic β-cell injury, hepatic inflammation, and the vascular, renal, and ocular complications of diabetes. Preclinical interruption of the circuit shows multi-organ benefit. Candidate strategies include peptidylarginine deiminase 4 inhibition, DNase I, NLRP3 blockade, and engineered therapeutic vesicles. Among clinically deployed agents, metformin and sodium–glucose cotransporter 2 inhibitors already show anti-trap and anti-inflammasome activity. The framework is mechanistically compelling. The evidence base, however, remains predominantly preclinical. No completed randomized controlled trial in obesity-related type 2 diabetes has yet validated it. Translation requires multidisciplinary collaboration, methodological standardization, sex-stratified design, and adequately powered prospective human studies.

## 1 Introduction

Obesity and type 2 diabetes mellitus (T2DM) are two of the most formidable public health crises of the 21st century. Approximately 589 million adults aged 20–79 were living with diabetes worldwide in 2024, a global prevalence of 11.1% (1). The figure is projected to reach 853 million by 2050, an increase of about 46% (1). Diabetes-related healthcare expenditure exceeds one trillion US dollars annually, and T2DM accounts for roughly 90–95% of all cases (1). The incidence of T2DM has risen by more than 73% over the preceding three decades, closely paralleling the global rise in obesity (2). The burden is disproportionately concentrated in low- and middle-income countries. These countries carry more than 80% of the global diabetes burden but have markedly limited access to standardized care (3). Urbanization, sedentary lifestyles, and energy-dense processed foods are the principal drivers of this “diabesity” epidemic. The sustained pressure on healthcare systems makes it urgent to clarify the underlying mechanisms and to develop effective interventions.

The mechanisms by which obesity promotes T2DM are multifaceted. Chronic low-grade inflammation is the pivotal pathological link. Adipose tissue expansion produces adipocyte hypertrophy, localized hypoxia, and oxidative stress. These changes activate the c-Jun N-terminal kinase (JNK) and nuclear factor-κB (NF-κB) signaling pathways. JNK/NF-κB activation polarizes macrophages toward the pro-inflammatory M1 phenotype. M1 macrophages secrete abundant TNF-α, IL-6, and IL-1β (4). These cytokines impair insulin receptor substrate (IRS) phosphorylation. They suppress the PI3K/Akt pathway and reduce GLUT4-mediated glucose uptake. The result is peripheral insulin resistance (5). Pro-inflammatory macrophages also infiltrate the pancreatic islets. There they activate the NLRP3 inflammasome and promote β-cell apoptosis, further compromising insulin secretion (6). The process extends beyond adipose tissue into systemic inflammation involving liver, skeletal muscle, and pancreas. Inflammation and metabolic dysfunction then reinforce each other (5). Chronic low-grade inflammation is therefore a critical entry point for understanding obesity-related T2DM and its multi-organ complications, and provides a clear rationale for anti-inflammatory therapeutic strategies (6).

Extracellular vesicles (EVs) are lipid bilayer-enclosed membrane structures secreted by virtually all cell types. They carry nucleic acids, proteins, and lipids, and serve as key mediators of intercellular and inter-organ communication (7). In the obese state, EVs from adipose tissue macrophages (ATMs) are enriched in pro-inflammatory microRNAs such as miR-155 and miR-34a. These EVs are delivered to neighboring adipocytes by paracrine mechanisms. There they suppress PPARγ expression and impair downstream insulin signaling (8). ATM-derived EVs also act directly on pancreatic β-cells. Through miR-155 they suppress glucose-stimulated insulin secretion and disrupt compensatory β-cell proliferation (9). The EV pool in obesity is not, however, uniformly pathogenic. Hepatocyte-derived exosomes from early-onset obese mice are enriched in miR-3075 and improve skeletal muscle insulin signaling (10). Small EVs from ATMs of rosiglitazone-treated obese mice (Rosi-ATM-sEVs) carry miR-690 and likewise improve insulin sensitivity (11). Intravenous administration of pathogenic ATM-derived EVs from obese mice to lean recipients induces impaired glucose tolerance, hyperinsulinemia, and insulin resistance (11). EVs can therefore transfer metabolic dysfunction across individuals. The pathogenic fraction of EVs is an important vehicle for the initiation and perpetuation of insulin resistance in obesity (12).

Neutrophil extracellular traps (NETs) are extracellular web-like structures released by activated neutrophils. They consist of decondensed chromatin scaffolds decorated with antimicrobial effector proteins. The principal constituents are myeloperoxidase (MPO), neutrophil elastase (NE), and citrullinated histone H3 (CitH3) (13). Neutrophils are among the earliest immune cells to infiltrate adipose tissue during high-fat diet-induced obesity. Metabolic signals such as free fatty acids and gut-derived lipopolysaccharide (LPS) translocation trigger NETosis (14). Circulating and adipose tissue NET markers, including cell-free DNA (cfDNA), MPO–DNA complexes, and CitH3, are elevated in obese individuals. Their levels correlate positively with the degree of metabolic dysregulation (15). Under T2DM conditions the hyperglycemic milieu further amplifies NETosis. The mechanism involves upregulation of GLUT1 in neutrophils, which enhances glucose uptake and glycolytic flux and promotes sustained NET accumulation in diabetic tissues (16). In diabetic atherosclerosis models NETs drive pro-inflammatory macrophage polarization within plaques and activate the NLRP3 inflammasome. They persist even after lipid reduction and thereby impede inflammatory resolution (17). Recent work additionally implicates neutrophil priming and trained immunity at the level of bone-marrow progenitors as drivers of persistent NET hyperactivity in chronic metabolic inflammation; we revisit this framework in §3 (18). NETs play a critical role in inflammatory amplification and complication progression in obesity-related T2DM.

EVs and NETs have each been investigated independently in metabolic disease. Their molecular interplay in obesity-driven T2DM remains insufficiently integrated. Two recent reviews have addressed adjacent territory. One described direct physical interaction between large EVs and NETs during systemic inflammation, with primary evidence drawn from sepsis and acute injury models (19). The other synthesized the shared effector molecules of neutrophil-derived EVs and NETs in canonical neutrophil biology (20). The present review extends this foundation in three ways. First, the focus is the obesity/T2DM metabolic microenvironment. The inflammatory drive here is chronic, low-grade, and sterile rather than acute. The change in context reshapes the EV–NET interaction. Second, the review integrates adipocyte- and ATM-derived EV cargo remodeling—including miR-155, miR-27a, miR-690, and miR-3075—with neutrophil priming and NETosis sensitization in metabolic disease. Neither prior review covered this angle. Third, the review maps organ-specific manifestations of the EV–NET axis across pancreatic islets, liver, kidney, vasculature, and adipose tissue. Each is linked to a clinically deployed or near-clinical intervention. Examples include metformin, sodium–glucose cotransporter 2 (SGLT2) inhibitors, the peptidylarginine deiminase 4 inhibitor GSK484, and engineered EV platforms. Throughout the manuscript we distinguish mechanisms directly demonstrated in obesity/T2DM from those inferred by analogy with sepsis, autoimmune, and oncological settings. The goal is a calibrated rather than over-extrapolated synthesis.

### 1.1 Handling of methodological heterogeneity during literature screening

The included studies employed heterogeneous methods for EV isolation (ultracentrifugation, size-exclusion chromatography, density-gradient, polymer precipitation, immunoaffinity capture) and NET quantification (MPO–DNA ELISA, CitH3 ELISA, immunofluorescence co-localization, cfDNA quantification, *in vivo* intravital imaging). To minimize bias arising from this heterogeneity, we applied the following data-extraction standards. (i) For each cited mechanistic claim, we recorded the EV isolation method and the panel of characterization markers reported (tetraspanins, size distribution, particle count, purity). Studies that did not meet at least the minimum MISEV2018/2023 reporting criteria (21, 22) were cited only for orientation purposes and not as primary mechanistic evidence. (ii) For NET-related claims, we required at least one orthogonal readout (e.g., MPO–DNA combined with CitH3, or biochemical assay combined with imaging) (23, 24). Single-marker (especially cfDNA-only) reports were treated as supportive rather than definitive. (iii) When mechanistic conclusions depended on a specific isolation/quantification platform, we explicitly flagged the methodological limitation in the relevant section (see §7.1 and §7.2). (iv) Conflicting findings across studies were interpreted in the context of their respective methods; where the discrepancy plausibly reflected method-driven bias (e.g., co-isolation of lipoproteins inflating “EV-associated” miRNA signals), this is stated in the text. We acknowledge that residual cross-study heterogeneity cannot be fully eliminated in a narrative synthesis, and that a future systematic review using MISEV2023-compliant primary data would strengthen quantitative integration.

### 1.2 Scope of this narrative review and literature search

This article is a narrative review, not a systematic review. It does not claim exhaustive coverage of the literature. It synthesizes representative mechanistic evidence on the EV–NET axis in obesity-induced T2DM and its multi-organ complications. The authors performed iterative searches of PubMed, Web of Science, and Scopus from inception through September 2025. Search terms included “extracellular vesicles,” “exosomes,” “neutrophil extracellular traps,” “NETosis,” “PAD4,” “obesity,” “insulin resistance,” “type 2 diabetes,” “sterile inflammation,” and “NLRP3.” Reference lists of key prior reviews (19, 20) and recent primary studies were hand-searched. Priority was given to mechanistic studies in mammalian metabolic disease models and human cohorts published in the last five years. Older landmark studies were retained where they established foundational concepts. Studies were excluded if they were non-peer-reviewed, not in English, or lacked mechanistic data relevant to the EV–NET interface. The manuscript is organized as follows: EV biology in obesity (§2); mechanisms of NET formation (§3); the EV–NET bidirectional feedback loop (§4); its role in driving obesity-induced T2DM and complications (§5); therapeutic strategies (§6); and current limitations and future perspectives (§§7 and 8).

This review examines the independent roles of EVs and NETs in obesity-related T2DM chronic inflammation. It elucidates the mechanisms underlying their interaction. It then explores potential therapeutic strategies and biomarker utility targeting the EV–NET axis. The review proceeds by first characterizing the biology of each system. It next addresses the interaction network through which the two systems cooperatively drive sterile inflammation. It then evaluates existing intervention evidence and closes with a perspective on emerging frontiers.

## 2 Biological basis of extracellular vesicles

### 2.1 Classification of extracellular vesicles

Extracellular vesicles (EVs) are small particles enclosed by a lipid bilayer, with diameters ranging from the nanometer to the micrometer scale. Nearly all cell types can secrete EVs, which primarily participate in intercellular material transfer and signal transduction (21). The International Society for Extracellular Vesicles (ISEV) provided detailed revisions to the nomenclature and characterization standards for EVs in the updated Minimal Information for Studies of Extracellular Vesicles (MISEV2023) (21). Given that current isolation technologies cannot yet fully resolve individual EV subpopulations, MISEV2023 therefore recommends describing EVs with operational terms based on physical or biochemical features, such as “small EVs (sEVs),” rather than inferential terms based on a specific biogenesis pathway (21).

According to biogenesis pathway and size, EVs can be divided into three major subtypes: exosomes (30–150 nm), microvesicles (MVs, 100–1,000 nm), and apoptotic bodies (ABs, 1–5 μm) (25). Exosomes are released through the endosomal pathway via fusion of multivesicular bodies (MVBs) with the plasma membrane, and their characteristic markers include the tetraspanins CD63, CD9, and CD81, as well as the biogenesis-associated proteins ALIX and TSG101 (26). MVs are formed by direct outward budding and shedding from the plasma membrane, and their characteristic markers are ARF6 and annexins A1 and A2 (27). Apoptotic bodies are generally produced by plasma membrane blebbing during programmed cell death and are rich in organelle fragments, nuclear material, and DNA; their characteristic markers include ROCK1, Pannexin-1, and externalized phosphatidylserine (PS) (28). As research has continued to advance, non-vesicular extracellular particles smaller than 50 nm, such as exomeres and supermeres, have also been identified in recent years; their biogenesis differs from that of classical EVs. Therefore, the overall composition of these extracellular particles is highly heterogeneous (29).

### 2.2 Biogenesis of EVs

#### 2.2.1 Biogenesis of exosomes

Exosome biogenesis can proceed through two pathways: ESCRT (endosomal sorting complexes required for transport)-dependent and ESCRT-independent (30).

The ESCRT-dependent pathway is regulated by four subcomplexes: HRS within ESCRT-0 recognizes ubiquitinated cargo and phosphatidylinositol-3-phosphate (PI3P) and recruits ESCRT-I (TSG101); ESCRT-II subsequently drives inward invagination of the endosomal membrane; ESCRT-III performs membrane neck scission; and finally, the VPS4 ATPase mediates the disassembly and recycling of ESCRT-III (30). Among these, the Syndecan–Syntenin–ALIX axis can recognize specific cargo (such as fibroblast growth factor receptors, FGFRs) and mediate the recruitment of ESCRT-III, ultimately promoting the formation of intraluminal vesicles (ILVs) (30).

The mechanisms involved in the ESCRT-independent pathway are more complex. For example, ceramide generated by neutral sphingomyelinase 2 (nSMase2) promotes ILV budding by inducing spontaneous membrane curvature; tetraspanin-enriched microdomains (TEMs) facilitate cargo sorting by clustering CD63, CD9, and CD81; Rab31 GTPase recruits flotillin proteins to ceramide- and cholesterol-enriched membrane microdomains and packages specific cargo into ILVs without relying on the ESCRT machinery or tetraspanins (31); Rab27a/b directly regulates the fusion of MVBs with the plasma membrane and exosome secretion. From a broader perspective, lipid overload caused by metabolic stress can impair lysosomal membrane integrity; the degradative route of MVBs is then compromised, and exosome release is favored instead (32).

#### 2.2.2 Biogenesis of microvesicles and apoptotic bodies

As mentioned above, MV biogenesis depends on disruption of plasma membrane phospholipid asymmetry and on cytoskeletal remodeling (33). Calcium influx or inflammatory stimulation can activate scramblase, inducing PS externalization to the outer membrane leaflet; subsequently, ARF6 drives the phospholipase D–ERK–myosin light chain kinase (MLCK) signaling cascade and further activates actomyosin-dependent contractile budding. In this process, the RhoA–ROCK axis is a key regulatory node. In the obese inflammatory microenvironment, the RhoA–ROCK axis is persistently activated, increasing MV secretion from adipocytes and immune cells (33). In contrast, apoptotic bodies are formed during caspase signaling by ROCK1-mediated plasma membrane blebbing; they contain nuclear debris and organelle remnants and are rapidly cleared by neighboring phagocytic cells (28).

### 2.3 Cargo composition of EVs

#### 2.3.1 Proteins

The EV proteome is composed of three categories of proteins. The first category is marker proteins, including the tetraspanins CD63, CD9, and CD81; the heat shock proteins HSP70 and HSP90; and the biogenesis-associated proteins ALIX and TSG101. The second category is parental cell-specific proteins, such as adipocyte-enriched perilipin A and fatty acid–binding protein 4 (FABP4). The third category is functional signaling proteins, such as cytokines, growth factors, and various enzymes (34). In adipose tissue–derived EVs (AT-EVs) from obese individuals, pro-inflammatory proteins such as retinol-binding protein 4 (RBP4) and osteoglycin (mimecan) are significantly enriched, and this enrichment can be regarded as one of the features of the pathological state of the parental cell (35).

#### 2.3.2 Nucleic acids

MicroRNAs (miRNAs, 19–24 nucleotides) are the most functionally characterized nucleic acid cargo in EVs. They primarily suppress target gene expression at the post-transcriptional level, and their core function is to bind to the 3′ untranslated regions (3′-UTRs) of mRNAs in recipient cells (35). The lipid bilayer of EVs protects miRNAs from degradation by circulating nucleases, ensuring their stable delivery to distal target organs (35). Selective packaging of miRNAs is co-regulated by the RNA-binding protein hnRNPA2B1 and the nSMase2 pathway; among these, hnRNPA2B1 recognizes specific sequence motifs, such as GGAG. These mechanisms determine whether a given miRNA is sorted into EVs or retained intracellularly, and this sorting is closely related to obesity-associated metabolic regulation (36). Meanwhile, EVs also mediate cross-cellular information transfer by transporting mRNAs, long non-coding RNAs (lncRNAs), circular RNAs (circRNAs), and others (37).

#### 2.3.3 Lipids

Exosomes are enriched in cholesterol, sphingomyelin, and ceramide; compared with the plasma membrane, their phosphatidylinositol content is relatively low (38). Lipids serve both as structural scaffolds and as bioactive signaling molecules. Obesity elevates the levels of sphingolipids (especially ceramide) and triglycerides in AT-EVs, and further activates the JNK and NF-κB inflammatory signaling pathways in recipient cells, mediating downstream lipotoxic effects (38).

### 2.4 Secretory characteristics of adipose tissue–derived EVs in obesity

Obesity remodels adipose tissue through pathways such as adipocyte hypertrophy, chronic inflammatory infiltration, and fibrosis, and further alters the secretion volume, size distribution, and cargo composition of AT-EVs (39).

#### 2.4.1 Changes in secretion volume and physical characteristics

In the obese state, AT-EV secretion is significantly upregulated. In adipose tissue samples from patients undergoing bariatric surgery, mature adipocytes secrete significantly more EVs than preadipocytes; meanwhile, pro-inflammatory stimuli (such as TNF-α and palmitate) further increase this release; and circulating EV counts in obese subjects correlate positively with BMI and with TNF expression in subcutaneous adipose tissue (SAT) (40). In fact, compared with SAT, visceral adipose tissue (VAT) secretes more EVs, and the quantity of VAT-derived EVs correlates positively with plasma triglyceride levels; this evidence indicates that the vesicles participate in systemic lipid redistribution (40). Interestingly, AT-EVs derived from lean and obese adipose tissue also differ functionally: obesity-derived AT-EVs exhibit enhanced pro-inflammatory activity and reduced protective signaling (41). In obese individuals, EVs derived from adipose-derived mesenchymal stem cells (ASCs) carry altered miRNA cargo, resulting in impaired anti-inflammatory and regenerative functions (42).

#### 2.4.2 Changes in pro-inflammatory miRNA cargo

In the obese state, the miRNA repertoire loaded into AT-EVs undergoes systematic remodeling, with continual enrichment of pro-inflammatory miRNAs:

miR-27a-5p: Significantly enriched in EVs from visceral adipocytes; its function is to suppress insulin secretion by pancreatic β-cells, resulting in impaired glucose tolerance (43).

**Figure 1:**
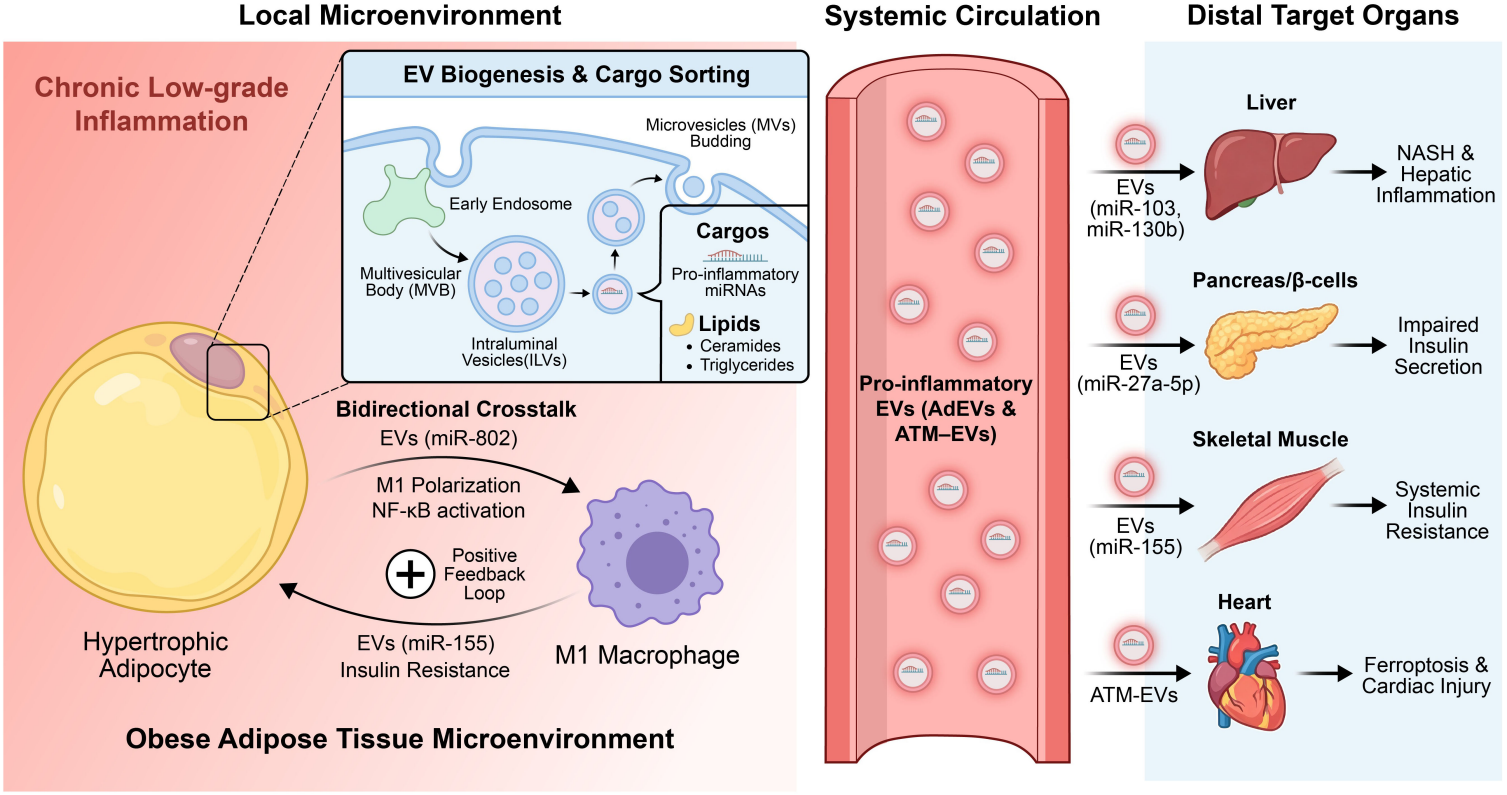
Extracellular vesicle (EV)–mediated local crosstalk and systemic multi-organ metabolic dysfunction in obesity. In the obese adipose tissue microenvironment (left panel), hypertrophic adipocytes and M1-polarized adipose tissue macrophages (ATMs) sustain a self-amplifying pro-inflammatory positive feedback loop. The loop operates through two unidirectional arms of EV-mediated communication. The adipocyte → ATM arm is mediated by adipocyte-derived EVs enriched in miR-802 and lipotoxic lipids (ceramides, triglycerides). These EVs drive ATM M1 polarization and NF-κB activation. The ATM → adipocyte/myocyte arm is mediated by ATM-derived EVs enriched in miR-155. These EVs reinforce local insulin resistance by suppressing *Pparg*. The closed loop is composed of two distinct directional arms. Each is carried by a different miRNA species. It is *not* a bidirectional flux of the same miRNA. During biogenesis, pro-inflammatory cargos are sorted into intraluminal vesicles (ILVs) within multivesicular bodies (MVBs) and released as exosomes. They may also be packaged into microvesicles via direct plasma membrane budding. The cargos include selected miRNAs, proteins, and bioactive lipids. Released into the systemic circulation (middle panel), these pathogenic EVs (AT-EVs and ATM-EVs) act as endocrine messengers. They deliver maladaptive miRNA cargo to distal metabolic organs (right panel). The outcomes are non-alcoholic steatohepatitis (NASH) and hepatic inflammation via miR-103 and miR-130b; impaired insulin secretion in pancreatic β-cells via miR-27a-5p; systemic insulin resistance via skeletal muscle delivery of miR-155; and cardiomyocyte ferroptosis and cardiac injury via ATM-EVs. The axis links adipose tissue chronic low-grade inflammation to multi-organ metabolic dysfunction. Schematic produced with BioRender.com (publication license obtained by the corresponding author). ATM, adipose tissue macrophage; AT-EVs, adipose tissue-derived EVs; ATM-EVs, ATM-derived EVs; EV, extracellular vesicle; ILV, intraluminal vesicle; MVB, multivesicular body; NASH, non-alcoholic steatohepatitis; NF-κB, nuclear factor-κB.

miR-802: Significantly enriched in EVs from obese adipose tissue. In the EV-mediated adipose–macrophage dialogue, miR-802 primarily carries the function of transmitting pro-inflammatory signals from adipocytes to ATMs: after transfer to macrophages via EVs, miR-802 directly activates the NF-κB signaling pathway, promoting adipose tissue inflammation and insulin resistance (44). It is worth noting that, as shown in **Figure 1**, miR-802 complements—rather than duplicates—the miR-155 axis through which ATMs transmit signals to adipocytes (see §2.5.1).

miR-130b: Transported by microvesicles from obese adipose tissue to the liver. In the liver, it activates hepatic inflammatory cascades by suppressing glucocorticoid receptor-mediated immunosuppression (45).

miR-103: Delivered by adipose tissue–derived exosomes to hepatocytes; by targeting PTEN, it activates the PI3K/AKT signaling pathway and accelerates the progression of non-alcoholic steatohepatitis (NASH) (46).

EV-loaded miRNAs can directly regulate biological processes such as browning of white adipose tissue, insulin resistance, and inflammation, and can be regarded as key drivers of obesity-associated metabolic diseases (47, 48). Obesity directly impairs EV function—for example, ASC-derived EVs from obese individuals show two changes: upregulation of NF-κB regulatory miRNAs and downregulation of protective miRNAs involved in angiogenesis and cell cycle regulation (42). Therefore, in obese mouse models, therapeutic interventions using EV-delivered miRNAs have demonstrated great translational potential (49).

#### 2.4.3 Changes in protein and lipid cargo

Adipocyte-derived exosomes can carry insulinotropic proteins that act directly on distal pancreatic β-cells, which can be regarded as a new route of inter-organ EV communication along the adipose–islet axis (50). Under metabolic stress, adipocytes also transfer mitochondria to macrophages via large EVs, directly participating in the regulation of adipose tissue energy metabolism (51).

Obesesomes are EVs shed from lipid-engorged hypertrophic adipocytes; they form a self-amplifying feedback cascade by stimulating macrophages to secrete pro-inflammatory cytokines such as TNF-α and IL-6 (52). As another example, brown adipose tissue (BAT)-derived EVs can carry miR-378a-3p and promote hepatic gluconeogenesis; after high-fat diet intervention, the metabolic-activity-related cargo profile of these EVs changes, demonstrating that obesity remodels the EV secretion profile of BAT (53, 54). Therefore, the circulating abundance of AT-EVs and the expression of adipocyte-specific proteins such as perilipin A can be regarded as potential biomarkers of adipocyte dysfunction and metabolic health (55).

### 2.5 Pro-inflammatory characteristics of immune cell–derived EVs in obesity

#### 2.5.1 Adipose tissue macrophage–derived EVs

Adipose tissue macrophages (ATMs) can secrete miRNA-containing EVs and regulate adipocyte function and the metabolism of distal organs through paracrine pathways (8). Obesity drives ATMs toward M1 polarization, and the EVs derived from these M1-polarized ATMs are enriched in pro-inflammatory miRNAs. Conversely, ATM-derived EVs induced by insulin-sensitizing drugs tend to carry anti-inflammatory miRNAs. These two types of EVs play opposite roles in the maintenance of metabolic homeostasis (56).

miR-155: Highly expressed in exosomes secreted by ATMs in obesity. Upon transfer to adipocytes and myocytes, miR-155 targets and regulates Pparg (mouse Pparg; human PPARG), inducing systemic insulin resistance and impaired glucose tolerance (39). In the obese adipose tissue microenvironment, the flux of miR-155 is unidirectional, being transmitted from M1-type ATMs to adipocytes and myocytes; conversely, the direction from adipocytes to ATMs is mediated by miR-802 (§2.4.2 and **Figure 1**) (44).

miR-690: Identified in small EVs released by adipose tissue macrophages of rosiglitazone-treated obese mice (Rosi-ATM-sEVs) (57, 58). It can directly enhance the insulin sensitivity of adipocytes, myotubes, and hepatocytes by suppressing Nadk, and inhibit inflammatory signaling; the donor macrophages exhibit M2-type features. It is worth noting that the M2 macrophages induced by rosiglitazone are not the classical IL-4-induced M2-type macrophages; therefore, when referring to this experimental source, the term “Rosi-ATM-sEVs” is generally used. Intravenous administration of Rosi-ATM-sEVs can reverse the obesity-induced glucose intolerance and insulin resistance in obese mouse models, and does not produce the weight gain and hemodilution commonly associated with rosiglitazone therapy (58). Therefore, miR-690-enriched Rosi-ATM-sEVs can be regarded as a next-generation therapeutic substrate for obesity-associated T2DM.

The pathological effects of ATM-derived EVs are not limited to local adipose tissue. Through the systemic circulation, these pro-inflammatory cargoes can reach tissues such as the liver, skeletal muscle, and pancreas, ultimately resulting in multi-organ metabolic dysfunction (59). For example, in the obese state, ATM-derived EVs reduce glutathione synthesis by suppressing SLC7A11, ultimately inducing ferroptosis of cardiomyocytes (60).

#### 2.5.2 EVs from adipose progenitor cells and bone marrow–derived cells

EVs derived from adipose progenitor cells (APCs) can directly regulate the immune function of adipose tissue. In middle-aged individuals, the level of miR-145-5p in APC-derived EVs is significantly decreased, leading to uncontrolled pro-inflammatory activation of ATMs and ultimately increasing susceptibility to midlife obesity. By restoring miR-145-5p levels, L-selectin expression in ATMs can be suppressed, antagonizing the M1 polarization program and alleviating adipose tissue inflammation (61). In addition, miR-6236 carried by EVs from bone marrow–derived myeloid cells can enhance insulin signaling in adipocytes and alleviate obesity-induced glycemic dysregulation (62). Within the adipose–islet metabolic communication network, these EVs can directly upregulate the compensatory adaptation of β-cells by influencing the crosstalk between ATMs and adipocytes (9).

#### 2.5.3 EV-mediated bidirectional regulatory network between adipocytes and immune cells

As shown in **Figure 1**, adipocytes and ATMs can engage in bidirectional regulation through EVs; however, different directions usually transport different miRNA cargoes. For example, the adipocyte → ATM direction is mainly driven by miR-802-enriched adipocyte EVs, with the function of inducing ATM polarization toward the M1 phenotype; the ATM → adipocyte/myocyte direction is driven by miR-155-enriched M1-ATM EVs, with the function of aggravating insulin resistance by suppressing PPARγ. These two unidirectional pathways close into a self-sustaining positive feedback loop of chronic inflammation. The network formed by this EV trafficking is a central molecular mechanism of obesity-associated metabolic inflammation, rather than a literal bidirectional flux of the same miRNA species (8, 44).

Section 2 summary. Obesity reshapes EV biology along three axes: elevated secretion volume; pro-inflammatory cargo enrichment (miR-155, miR-27a-5p, miR-802, miR-130b, RBP4, ceramide); and depletion of protective miRNAs (miR-690 in Rosi-ATM-sEVs, miR-145-5p in APC-EVs, miR-6236 in bone-marrow EVs). The result is summarized in **Figure 1**. The “pro-inflammatory cargo remodeling” underlies multi-organ metabolic dysregulation and motivates the EV-targeted therapeutic strategies detailed in §6 (11, 63–65).

## 3 Mechanisms of neutrophil extracellular trap formation

### 3.1 Concept and biological characteristics of NETs

Neutrophils are the most abundant leukocytes in the peripheral circulation. They are core effector cells of the innate immune defense system. NETs were first described in 2004. Activated neutrophils release a web-like extracellular structure composed of decondensed chromatin DNA and antimicrobial granule proteins; this structure captures and kills pathogens (66). The discovery reshaped the conceptual framework of innate immunology regarding neutrophil function (67).

NETs are multimolecular reticular structures. They are composed of highly decondensed chromatin DNA scaffolds and diverse functional proteins. The principal components are histones (particularly CitH3); granule-derived serine proteases including NE and proteinase 3 (PR3); MPO; the antimicrobial peptide LL-37/cathelicidin; and cytosolic proteins such as high-mobility group box 1 (HMGB1) and calprotectin (S100A8/A9) (68). Individual NET fibers are approximately 15–17 nm in diameter. They form capturing networks spanning hundreds of square micrometers both *in vitro* and *in vivo*. These networks effectively restrict pathogen dissemination (66).

The process of NET formation is termed NETosis. It is a specialized form of programmed cell death distinct from classical apoptosis and necrosis (69). Under physiological conditions NETs constitute a critical innate immune defense against bacterial, fungal, and viral infections. Under pathological conditions including obesity, hyperglycemia, and hyperlipidemia, sustained overproduction of NETs disrupts immune homeostasis. The consequences include vascular endothelial injury, thrombosis, and immunopathological reactions. NETs are therefore a key pathological driver of multi-organ metabolic dysfunction and cardiovascular injury (70).

### 3.2 Molecular mechanisms of NETosis

NETosis is classified into two major subtypes by dependence on reactive oxygen species (ROS) and by whether neutrophil death accompanies the process. The two subtypes are ROS-dependent suicidal NETosis and ROS-independent vital NETosis. Several variant mechanisms have also been characterized (69).

#### 3.2.1 ROS-dependent NETosis (suicidal NETosis)

Classical ROS-dependent NETosis unfolds over 3–8 hours. It culminates in neutrophil death. Diverse stimuli induce it, including phorbol 12-myristate 13-acetate (PMA), lipopolysaccharide (LPS), and immune complexes (71).

##### 3.2.1.1 Receptor recognition and signal cascade initiation

Exogenous or endogenous stimuli bind to neutrophil surface pattern recognition receptors (PRRs). These include TLR1/2/4/7/9 and Fc receptors. PRR engagement activates the downstream PKC–Raf–MEK–ERK signaling cascade. It also triggers the assembly and activation of NADPH oxidase (NOX2) (67). Fully activated NOX2 catalyzes the reduction of molecular oxygen to superoxide anion (O_2_^-^). O_2_^-^ is then converted to H_2_O_2_ by superoxide dismutase (SOD). The product is the characteristic neutrophil oxidative burst (67). Patients with chronic granulomatous disease (CGD) harbor NOX2 mutations that ablate NADPH oxidase function. Their neutrophils are incapable of PMA-induced classical suicidal NETosis. This provides genetic evidence for an indispensable role of NOX2 in this pathway (71).

##### 3.2.1.2 Granule protein nuclear translocation and histone degradation

Driven by ROS, NE and MPO are released from azurophilic granules into the cytoplasm. NE first proteolytically degrades F-actin and other cytoskeletal proteins. It then translocates to the nucleus. In the nucleus it collaborates with MPO to partially hydrolyze histone H1. The interaction force between DNA and histones is thereby reduced, initiating chromatin decompaction (68).

##### 3.2.1.3 PAD4-mediated histone citrullination and chromatin decondensation

Peptidylarginine deiminase 4 (PAD4) is the pivotal enzymatic hub for global chromatin decompaction. PAD4 is activated upon elevation of intracellular Ca²^+^. After nuclear translocation it catalyzes the irreversible citrullination of arginine residues on histones H3 and H4 (72). Citrullination neutralizes the positive charge of arginine and weakens electrostatic interactions between histones and DNA. Highly condensed chromatin can then unfold (72). Cit-H3 serves as a specific circulating biomarker for excessive NET release. It is significantly elevated in patients with obesity, T2DM, and atherosclerosis (73).

##### 3.2.1.4 Nuclear envelope and plasma membrane rupture, and NET release

NOX2-derived ROS drive protease-mediated cleavage of gasdermin D. The N-terminal fragment (GSDMD-NT) forms non-selective pores in the plasma membrane. NE leakage from granules and nuclear envelope rupture follow (74). Decondensed chromatin mixes with granule contents and diffuses into the cytoplasm. It is then expelled into the extracellular space through explosive cell lysis (74).

#### 3.2.2 ROS-independent NETosis (vital NETosis)

Vital NETosis is completed within 5–60 minutes. It occurs independently of NOX2 activation and without immediate neutrophil death. Nuclear chromatin is selectively packaged into budding vesicles and released extracellularly via exocytosis. The neutrophil retains intact plasma membrane and effector functions such as phagocytosis (69).

##### 3.2.2.1 The pivotal role of mitochondrial ROS

The key signal driving vital NETosis is mitochondria-derived ROS (mROS), not cytosolic NOX2-derived ROS (75). Elevation of intracellular Ca²^+^ activates the small-conductance calcium-activated potassium channel SK3 (KCNN3) on the mitochondrial membrane. The result is mitochondrial membrane potential (ΔΨm) hyperpolarization, increased electron leakage from the electron transport chain, and mROS accumulation (75). mROS independently activates PAD4-mediated histone citrullination and drives vesicular nuclear DNA extrusion. Ca²^+^ influx can also induce opening of the mitochondrial permeability transition pore (mPTP), releasing mitochondrial DNA (mtDNA) to form a distinct mitochondrial NET subtype (75).

##### 3.2.2.2 Platelet activation-induced vital NETosis

Activated platelets promote rapid NOX2-independent NET release within minutes. The signals are platelet-activating factor (PAF) and HMGB1, or direct contact between P-selectin and neutrophil PSGL-1 (70). This platelet–neutrophil axis-mediated NETosis plays important pathological roles in venous thrombosis and metabolic inflammation (70).

#### 3.2.3 Additional NETosis pathways

##### 3.2.3.1 NLRP3 inflammasome-dependent NETosis

PAD4 promotes NLRP3 inflammasome assembly by upregulating NLRP3 and ASC protein expression. The result is caspase-1 activation. Caspase-1 then mediates nuclear membrane expansion and plasma membrane rupture through the GSDMD pore mechanism, driving NET release (72). Pharmacological inhibition of NLRP3 (e.g., MCC950) significantly reduces NET formation. It exerts protective effects in *in vivo* thrombosis models (72).

##### 3.2.3.2 Necroptosis pathway (RIPK3–MLKL axis)

Activation of the RIPK3–MLKL signaling axis causes MLKL to accumulate at the neutrophil plasma membrane. MLKL forms non-selective ion channels. Na^+^ influx induces cell swelling. The already decondensed chromatin is then released as NETs following plasma membrane rupture. This pathway contributes to infectious necrosis and metabolic stress-related tissue injury (69).

### 3.3 Triggering signals and activation conditions for NETs

NET release is the final innate immune effector output following neutrophil integration of multiple danger signals. It occurs under the following major conditions (13).

#### 3.3.1 Pathogen-associated molecular patterns and damage-associated molecular patterns

Bacteria (Gram-positive and Gram-negative), fungi (*Candida albicans*, *Aspergillus*), and viruses activate neutrophils through TLR1/2/4/7/9 and NOD1/2. NETosis is then initiated (67). Endogenous DAMPs trigger NET release through NLRP3 inflammasome activation and TLR recognition. The principal DAMPs include HMGB1, uric acid crystals, cholesterol crystals, ox-LDL, mtDNA, and extracellular nucleosomes. They play particularly important roles in non-infectious metabolic inflammation (76).

#### 3.3.2 “Priming” effects of pro-inflammatory cytokines

TNF-α, IL-1β, IL-8 (CXCL8), and granulocyte-macrophage colony-stimulating factor (GM-CSF) prime neutrophils. They lower the threshold for subsequent NETosis activation without independently triggering NET formation (77). In obesity, sustained hypersecretion of these cytokines by adipose tissue maintains circulating neutrophils in a persistently primed state, substantially reducing the NETosis activation threshold (15). The functional consequences of peripheral priming—and its extension into bone-marrow-level epigenetic reprogramming (“trained immunity”)—are discussed in §3.6.

#### 3.3.3 Platelet activation products and coagulation system components

Activated platelets release PAF and HMGB1. Coagulation and complement activation produces thrombin, fibrin, and complement C5a. All of these activate neutrophils through their respective receptors and induce vital or suicidal NETosis (70).

#### 3.3.4 Metabolic products and lipid signaling molecules

Advanced glycation end-products (AGEs), oxidized low-density lipoprotein (ox-LDL), saturated free fatty acids (FFAs), and cholesterol crystals directly activate neutrophil NETosis. The receptors involved are TLR4, RAGE, scavenger receptor CD36, and NLRP3. These ligands are the core molecular drivers of excessive NET release in metabolic syndrome (73).

### 3.4 Promotion of NET formation by obese, hyperglycemic, and hyperlipidemic microenvironments

Obesity, hyperglycemia, and hyperlipidemia create a sustained systemic pro-inflammatory microenvironment. This microenvironment enhances neutrophil NETosis sensitivity across multiple dimensions (73).

#### 3.4.1 Obese microenvironment and neutrophil NETosis

Neutrophils are among the earliest immune cells to infiltrate visceral adipose tissue (VAT) following high-fat diet (HFD)-induced obesity. They accumulate extensively in VAT during early obesity. They are the initial triggering cell population for chronic adipose tissue inflammation.

##### 3.4.1.1 Pro-NETosis effects of adipokines

Obesity causes adipose tissue to aberrantly secrete large quantities of pro-inflammatory adipokines. Leptin activates downstream signaling pathways by binding to the LepRb receptor on neutrophils. This upregulates NOX2 subunit expression. Neutrophil ROS production capacity and spontaneous NETosis rates increase significantly (15). Resistin and visfatin (NAMPT) further augment NETosis propensity by activating the TLR4–NF-κB axis (15).

##### 3.4.1.2 Adipose tissue hypoxia-driven NETosis

The markedly reduced local oxygen tension in over-expanded adipose tissue stabilizes hypoxia-inducible factor-1α (HIF-1α) in neutrophils. mROS generation increases. Neutrophils shift toward a NETosis-primed state (70).

##### 3.4.1.3 Clinical evidence

Obese subjects exhibit significantly higher plasma MPO–DNA complex and Cit-H3 concentrations than normal-weight controls. NET biomarker levels correlate positively with BMI and adipose tissue inflammation (15). In HFD-fed mouse models, NET deposition in mesenteric arteriole walls is associated with endothelial dysfunction. Treatment with the PAD4 inhibitor Cl-amidine or with DNase restores endothelial function. NETs are therefore a critical mediator of obesity-associated vascular injury (78).

#### 3.4.2 Hyperglycemic (diabetic) microenvironment and excessive NET release

Hyperglycemia substantially potentiates neutrophil NETosis through multiple independent mechanisms. It is the primary driver of systemic NET overactivation in diabetic patients (79).

##### 3.4.2.1 NOX2 hyperactivation and enhanced oxidative burst

Hyperglycemia upregulates NOX2 subunits gp91phox and p47phox via PKC-βII activation. *In vitro*, increasing glucose concentration from 5.5 mM to 25 mM elevates both the NETosis rate and NOX2-derived ROS generation in a concentration-dependent manner (80). T2DM patients show elevated plasma levels of NE, mono/oligonucleosomes, and cell-free double-stranded DNA. These levels correlate positively with HbA1c (80).

##### 3.4.2.2 AGE–RAGE axis activation of NETosis

AGEs accumulating under diabetic conditions bind to the RAGE receptor on neutrophils. The MAPK–ERK signaling cascade is activated. Ca²^+^ influx facilitates PAD4 activation. NLRP3 inflammasome protein levels are upregulated. The AGE–RAGE axis thus concurrently promotes ROS-dependent and ROS-independent NETosis (79).

##### 3.4.2.3 NET–NLRP3–IL-1β positive feedback loop

In diabetic kidney disease (DKD), NETs activate the NLRP3 inflammasome in glomerular endothelial cells under high-glucose conditions. IL-1β is produced. IL-1β in turn stimulates further neutrophil NETosis. The self-amplifying NET–NLRP3–IL-1β–NET loop is a key mechanism driving the progression of diabetic microvascular complications (76).

##### 3.4.2.4 PAD4–NLRP3 axis in diabetic organ injury

In diabetic kidney disease models, NETs promote renal fibrosis and IL-1β release. The mechanism involves activation of the NLRP3 inflammasome and PAD4-dependent pathways in glomerular endothelial cells. PAD4 inhibition significantly attenuates this injury process. The PAD4–NLRP3 pathway is a core hub for high glucose-driven NET-mediated organ damage (76).

##### 3.4.2.5 Impaired NET clearance

Hyperglycemia not only promotes NET release but also impairs NET degradation. Circulating DNase I activity is significantly reduced in diabetic patients. High glucose also inhibits macrophage efferocytosis capacity. NETs accumulate in tissues and prolong injury (79).

#### 3.4.3 Hyperlipidemia and NET activation

Plasma triglyceride, LDL, and FFA levels correlate positively with circulating NET biomarker concentrations. Hyperlipidemia is therefore an independent metabolic risk factor for neutrophil NETosis (73).

##### 3.4.3.1 Oxidized low-density lipoprotein

Ox-LDL induces concentration- and time-dependent ROS generation and NET release in neutrophils. The primary mechanism is synergistic activation of scavenger receptor CD36 and TLR2/TLR6. This engages the TLR–PKC–IRAK–MAPK signaling cascade and activates NOX. NOX inhibitors and TLR2/6-blocking antibodies significantly suppress this effect (73).

##### 3.4.3.2 Cholesterol crystals

Cholesterol crystals trigger neutrophil ROS bursts and NE nuclear translocation, inducing NET release. In atherosclerotic mouse models NETs further activate plaque macrophage NLRP3 inflammasomes. IL-1β and IL-18 are released. A lipid–NET–inflammasome positive feedback loop drives atherogenesis. NE/PR3 double knockout or DNase treatment significantly reduces plaque size and IL-1β levels (76).

##### 3.4.3.3 Free fatty acids

Saturated FFAs, particularly palmitic acid, activate neutrophils via the TLR4–MyD88–NF-κB pathway. NOX2 expression is upregulated. ROS production is augmented. Mitochondrial dysfunction with mROS bursts develops. Spontaneous NETosis rates rise significantly (73). n-3 polyunsaturated fatty acids (EPA and DHA) partially antagonize saturated FFA-induced NETosis. They do so through GPR120 (FFAR4) receptor activation and competitive inhibition of TLR4 ligand binding. Fatty acid type and saturation are therefore important metabolic variables governing NETosis propensity (73).

#### 3.4.4 Sex differences in NETosis and EV biology in metabolic diseases

Sex is an important modulator of metabolic inflammation, and consequently most research on metabolic diseases has relied on male animals or mixed-sex human cohorts. Under HFD/streptozotocin (STZ) treatment conditions, for example, male C57BL/6J mice develop pronounced hyperinsulinemia and insulin resistance, whereas female mice do not exhibit these phenotypes. The presence of estrogen substantially modifies the metabolic characteristics of these mice, such that data derived from male mice cannot be extrapolated to the pathological context of human females (81).

Sex factors also shape neutrophil biology. Estrogen suppresses PAD4 expression and further inhibits NADPH oxidase activity; accordingly, neutrophils from pre-menopausal women exhibit lower levels of both spontaneous and stimulated NETosis compared with age-matched men, while the post-menopausal decline in estrogen levels may increase the risk of cardiometabolic disease in post-menopausal women (82). As described previously (§3.3), sex hormones modulate the cytokine-priming environment that lowers the NETosis threshold: estradiol suppresses TNF-α and IL-6 production by adipose tissue macrophages, whereas androgens promote a pro-inflammatory adipose tissue immune milieu that primes neutrophils for NET release (82).

Sex also influences the adipose tissue EV profile. Under normal conditions, men harbor greater amounts of visceral adipose tissue and secrete more inflammatory mediators. Correspondingly, the proportion of EVs carrying pro-inflammatory miRNAs (miR-155, miR-27a) released from male visceral adipose tissue is markedly higher than that from the subcutaneous adipose tissue predominating in women. However, pre-menopausal women bear a substantially lower risk of obesity-related cardiometabolic disease than age-matched men (39, 83). It can therefore be inferred that the post-menopausal decline in estrogen levels is an important contributor to the elevated cardiometabolic disease risk subsequently observed in women. In the NOD mouse model, female animals exhibit higher islet neutrophil elastase activity and NET deposition than their male counterparts. Collectively, these findings suggest that EVs represent an important mediator of sex–disease interactions and may further complicate disease progression and its clinical manifestations (84). Future mechanistic studies of EV–NET interactions and related therapeutic research should therefore report sex-stratified data, and both treatment windows and biomarker thresholds should explicitly account for sex-based differences.

### 3.5 Key NET components and their tissue injury mechanisms

NETs carry a diverse array of bioactive molecules, several of which inflict systemic damage on vascular endothelium, parenchymal organs, and stromal tissues through mechanisms encompassing direct cytotoxicity, proteolytic activity, oxidative damage, and immunogenic activation (13).

#### 3.5.1 Direct toxicity and immune activation by the extracellular DNA scaffold

High concentrations of double-stranded DNA (dsDNA) can directly activate TLR9 within endosomal compartments of endothelial cells, engaging the MyD88–NF-κB pathway and accelerating the release of inflammatory mediators including IL-6, IL-8, and TNF-α (67). Cell-free DNA (cfDNA) constitutes a central component of NETs; NET-derived cfDNA additionally activates the cGAS–STING innate immune pathway, driving a robust type I interferon (IFN-α/β) response that promotes autoimmune inflammation and tissue injury (77). Within the vasculature, DNA–histone complexes bind platelets directly, triggering platelet aggregation and the coagulation cascade and thereby promoting thrombotic microangiopathy.

Clinically, MPO–DNA complexes can be used to directly quantify systemic NET burden. As discussed in §7.2, antibody specificity, calibrator stability, and cross-interference from non-NET DNA sources all affect assay performance (23, 70). To achieve quantitative accuracy, clinical measurement of MPO–DNA levels should therefore be corroborated by at least one additional readout, such as citrullinated histone H3 (Cit-H3) ELISA or imaging-based co-localization, to ensure the reliability of the final results.

#### 3.5.2 Cytotoxic effects of extracellular histones

Extracellular histone H4 has a highly cationic surface. It directly binds negatively charged phospholipids in target cell membranes. It inserts into the lipid bilayer and forms transmembrane pores. The result is intracellular Ca²^+^ overload and lytic cell death (85). In atherosclerotic mouse models, extracellular histone H4 binds to and lyses smooth muscle cells. It drives arterial tissue damage and sustains chronic inflammation. Neutralization of histone H4 prevents smooth muscle cell death and stabilizes atherosclerotic plaques (85). Extracellular histones also activate platelet TLR2/TLR4. The result is platelet activation and a NET–histone–platelet aggregation–thrombosis amplification cascade (70). Circulating Cit-H3 levels are closely associated with the degree of cardiac and renal injury in obese and diabetic patients. The marker therefore has significant biomarker value (73).

#### 3.5.3 Neutrophil elastase and proteinase 3

During NETosis, NE first translocates to the nucleus. It participates in limited histone hydrolysis and chromatin decondensation. NE is a critical enzymatic driver of suicidal NETosis. Extracellularly released NE subsequently degrades extracellular matrix (ECM) proteins including elastin, fibronectin, laminin, and type IV collagen. The result is tissue structural disruption and loss of basement membrane integrity (68).

PR3 belongs to the azurophilic granule serine proteases, as does NE. It degrades various ECM components. It also generates fully bioactive pro-inflammatory cytokines by cleaving IL-1β and TNF-α precursors. PR3 thus enzymatically amplifies the breadth and intensity of inflammatory responses (13).

#### 3.5.4 Myeloperoxidase and other functional components

Within NETs, MPO exists in the form of MPO–DNA complexes; As noted in §3.5.1, this complex serves as the most widely used—rather than gold-standard—clinical surrogate for systemic NET release (23, 70). Extracellular MPO exploits H_2_O_2_ to oxidize Cl^-^ and generate hypochlorous acid (HOCl). HOCl disrupts tight junction proteins in endothelial cells, increases vascular permeability, and ultimately impairs endothelial barrier function (13). Under conditions of obesity and diabetes, the oxidative activity of MPO further activates the NLRP3 inflammasome, establishing an amplification loop for metabolic inflammation (72).

NETs also carry a variety of additional bioactive molecules. HMGB1 functions as a DAMP-mediated amplifier of inflammation: it activates the TLR2/TLR4/RAGE axis and suppresses macrophage efferocytosis, thereby prolonging the pathological persistence of NETs at sites of injury (13). LL-37/cathelicidin forms immunogenic complexes with NET-associated DNA, activating plasmacytoid dendritic cells (pDCs) to produce IFN-α and sustaining autoimmune inflammation (77). Tissue factor (TF) is expressed on the NET surface and initiates the extrinsic coagulation pathway (the FVIIa–TF–FXa cascade), promoting microthrombosis in obesity- and metabolic syndrome-related thrombotic events (70). Calprotectin (S100A8/A9) activates TLR4 and RAGE on monocytes, inducing secretion of TNF-α and IL-6 and cascading amplification of the downstream systemic inflammatory response elicited by NETs (68).

### 3.6 Neutrophil priming and trained immunity in chronic metabolic inflammation

Neutrophils in obesity and T2DM undergo two functionally distinct forms of long-lived reprogramming, beyond the acute receptor-driven NETosis programs detailed above. Together these forms explain the *persistence* of NET hyperactivity in chronic metabolic disease.

#### 3.6.1 Peripheral priming

A sub-activating exposure to GM-CSF, TNF-α, IL-8, leptin, or hyperglycemia-derived AGEs lowers the NETosis activation threshold without independently triggering NET release (77). Each of these signals is chronically elevated in obesity/T2DM. Primed circulating neutrophils in obese individuals respond to subsequent triggers (LPS, ox-LDL, immune complexes) with disproportionately large NET output (15, 77). Peripheral priming thus translates the chronic adipose-tissue cytokine milieu into systemic NETosis hypersensitivity. Priming is *reversible* within hours-to-days upon withdrawal of the priming signal. It is therefore mechanistically distinct from the longer-lived trained-immunity programs described next.

#### 3.6.2 Trained immunity

Trained immunity was originally defined in monocyte–macrophage biology. It has more recently been extended to the granulocytic lineage and to hematopoietic progenitors in the bone marrow (18). The mechanism involves epigenetic and metabolic rewiring at the progenitor level. H3K4me3 and H3K27ac accumulate at pro-inflammatory loci. A metabolic shift toward glycolysis and accumulation of fumarate and mevalonate occurs. Successive cohorts of innate immune cells with a heightened pro-inflammatory phenotype are then generated. This phenotype persists across cell generations (18). A four-week Western diet in *Ldlr*^–/–^ mice triggers NLRP3-dependent reprogramming of bone-marrow myeloid progenitors (86). The trained-immunity signature is maintained for at least four weeks after reversion to chow diet. Differentiated myeloid cells remain hyperresponsive to subsequent TLR stimulation (86). In atherosclerosis-prone models and in human metabolic syndrome, ox-LDL, β-glucan, oxidized phospholipids, and saturated fatty acids drive analogous granulopoietic reprogramming. Successive cohorts of neutrophils with hair-trigger NETosis emerge, even after the original metabolic insult is withdrawn (87).

#### 3.6.3 Clinical implications

This framework helps explain three otherwise puzzling clinical observations in obesity/T2DM. First, elevated circulating MPO–DNA and Cit-H3 persist after substantial weight loss, including post-bariatric surgery. Second, cardiovascular event rates fall over the long term after only short bariatric-induced weight loss, more than acute metabolic improvement alone can explain. Third, early glycemic control has a “legacy effect” on later complications. The framework also predicts that *peripheral* anti-NET interventions (DNase I, PAD4 inhibitors administered systemically) may underperform unless bone-marrow-level reprogramming is also addressed. Agents that modulate myeloid-progenitor epigenetic state—methylation/acetylation inhibitors, IL-1β neutralization, mevalonate-pathway modulators—are therefore worth exploring as complementary strategies. We return to this concept in the therapeutic discussion (§6.2 and §6.6.3).

**Figure 2:**
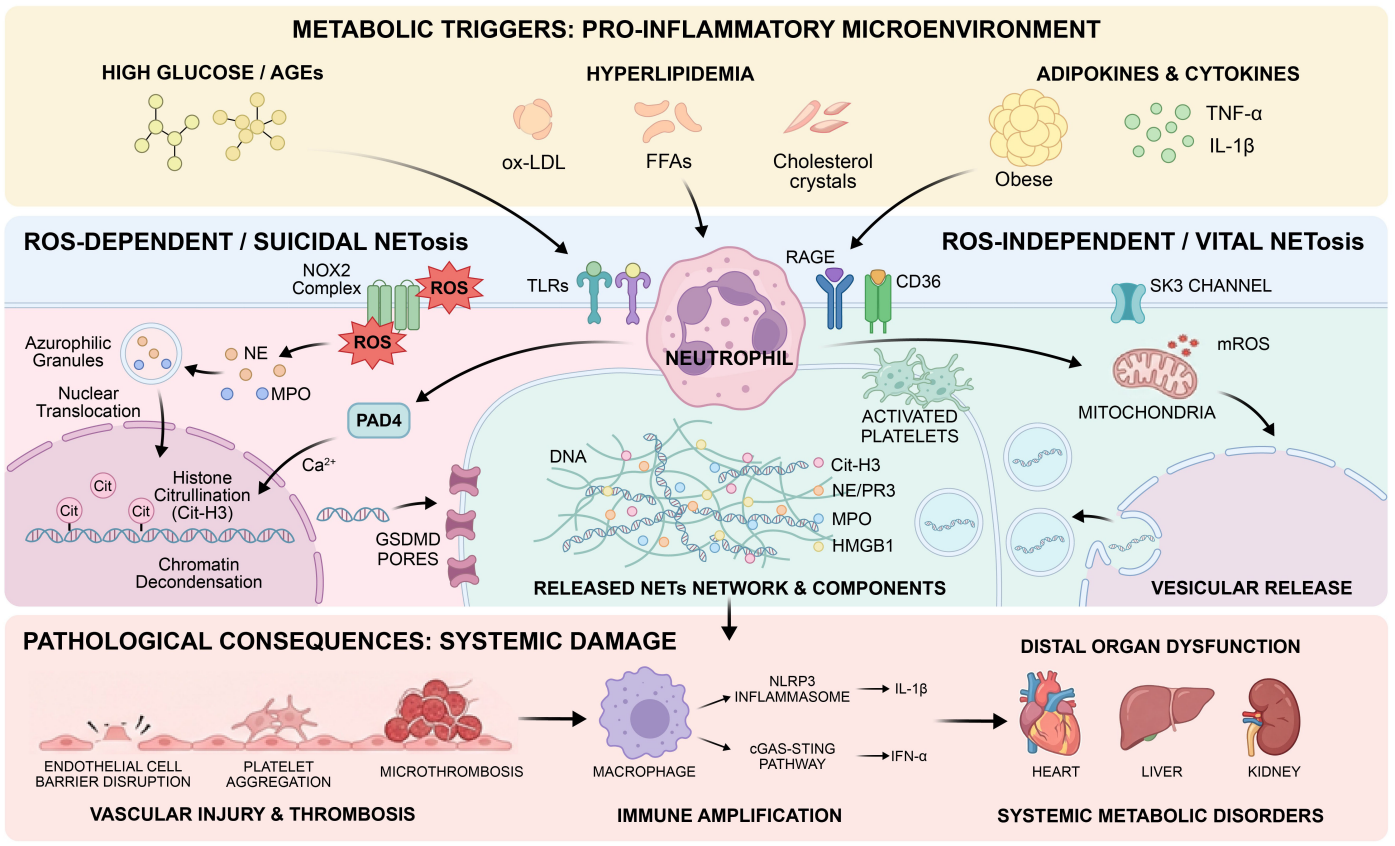
Metabolic microenvironment–driven mechanisms of NETosis and their systemic pathological consequences. Under conditions of obesity, hyperglycemia, and hyperlipidemia (top panel), metabolically derived danger signals engage neutrophil surface receptors. The danger signals include AGEs, ox-LDL, FFAs, cholesterol crystals, adipokines, and cytokines (TNF-α, IL-1β). The receptors include TLRs, RAGE, and CD36. Two mechanistically distinct programs of NET formation are then activated. Left, ROS-dependent suicidal NETosis: NOX2-derived ROS trigger release of NE and MPO from azurophilic granules. Nuclear translocation follows. Ca²^+^-dependent PAD4 activation then drives histone H3 citrullination (Cit-H3) and chromatin decondensation. GSDMD pore–mediated plasma membrane rupture and explosive NET extrusion complete the program. Right, ROS-independent vital NETosis: Mitochondrial ROS (mROS) are generated downstream of SK3 channel activation. Together with platelet–neutrophil interactions, mROS drive vesicular DNA exocytosis. Plasma membrane integrity is preserved. The released NET meshwork (middle) is decorated with cfDNA, Cit-H3, NE/PR3, MPO, and HMGB1. Bottom panel, systemic damage: NET components disrupt the endothelial barrier. Platelet aggregation and microthrombosis are promoted. Sterile inflammation is amplified through macrophage NLRP3 inflammasome–IL-1β and cGAS-STING–IFN-α signaling. Injury is then propagated to the heart, liver, and kidney. Systemic metabolic disorders are aggravated. Schematic produced with BioRender.com (publication license obtained by the corresponding author). AGEs, advanced glycation end-products; cfDNA, cell-free DNA; Cit-H3, citrullinated histone H3; FFAs, free fatty acids; GSDMD, gasdermin D; HMGB1, high-mobility group box 1; MPO, myeloperoxidase; mROS, mitochondrial ROS; NE, neutrophil elastase; NET, neutrophil extracellular trap; NOX2, NADPH oxidase 2; ox-LDL, oxidized low-density lipoprotein; PAD4, peptidylarginine deiminase 4; PR3, proteinase 3; RAGE, receptor for advanced glycation end-products; ROS, reactive oxygen species; SK3, small-conductance calcium-activated potassium channel 3; TLR, Toll-like receptor.

As summarized in **Figure 2**, NETosis is a multi-program effector output. It integrates ROS-dependent (NOX2 → NE/MPO → PAD4 → GSDMD) and ROS-independent (mROS, platelet, NLRP3, RIPK3–MLKL) pathways. All of these are amplified in the obese/diabetic milieu through adipokine-, AGE–RAGE-, ox-LDL/FFA-, and impaired-clearance-dependent mechanisms. Sex (§3.4.4) and trained-immunity-based progenitor reprogramming (§3.6) add two further axes of regulation. These have been historically under-recognized in metabolic NET research. Both are essential for a balanced view of NET biology in obesity-related T2DM.

## 4 The Interaction between EVs and NETs: a bidirectional pro-inflammatory loop

### 4.1 Molecular mechanisms by which EVs promote neutrophil activation and NET release

Extracellular vesicles (EVs) carry a diverse array of bioactive molecules. The cargo includes lipid mediators, proteins, and nucleic acids. EVs deliver this cargo to target cells through membrane fusion or endocytic pathways. They are essential mediators of intercellular communication (88). EVs derived from activated platelets, endothelial cells, and immune cells participate in neutrophil activation. They exert critical regulatory roles in inflammatory diseases (7).

Pathogen-associated molecular patterns (PAMPs) or damage-associated molecular patterns (DAMPs) stimulate neutrophils to undergo NETosis. NETosis produces web-like extracellular structures composed of chromatin DNA, histones, and granule proteins. The principal proteins are neutrophil elastase (NE), myeloperoxidase (MPO), and citrullinated histone H3 (CitH3) (7).

#### 4.1.1 Note on evidence provenance

Several mechanistic principles in this subsection have been most rigorously demonstrated in acute inflammatory contexts (sepsis, COVID-19), in autoimmune disease, or in cancer rather than in obesity/T2DM. They are summarized here because the signaling logic is highly likely to be conserved. Readers should keep this provenance in mind. We recapitulate the obesity/T2DM-direct evidence explicitly in §4.5.

Platelet-derived EVs (PEVs) are internalized by neutrophils through the synergistic action of 12-lipoxygenase (12-LO) and secretory phospholipase A2-IIA (sPLA2-IIA), with the lipid mediator 12(S)-HETE serving as the key endocytic signal (89). Upon sensing lipopolysaccharide (LPS) and other TLR4 ligands, platelet TLR4 binds to neutrophils and induces NET formation, a mechanism critical for intravascular bacterial trapping during sepsis (90). In patients with rheumatoid arthritis, PEV concentrations are markedly elevated in synovial fluid, and these IL-1–rich microparticles amplify local pro-inflammatory signaling within the joint by activating synovial fibroblasts (91).

Under hypoxia or pro-inflammatory cytokine stimulation, activated endothelial cells release endothelial-derived EVs (EEVs). These EEVs are enriched in tissue factor (TF), von Willebrand factor (vWF), and intercellular adhesion molecule-1 (ICAM-1) (92). EEVs activate the Ca²^+^/protein kinase C (PKC) signaling pathway in neutrophils; PAD4 subsequently translocates to the nucleus and becomes activated, further catalyzing histone H3 citrullination and driving chromatin decondensation and NET formation (8). Although this is not a systematic investigation in metabolic disease, it nonetheless illustrates the conserved signaling cascade of NET formation: in critically ill COVID-19 patients, extensive NET formation drives immunothrombosis and produces widespread multi-organ injury (93). In the tumor microenvironment, tumor cells continuously release granulocyte colony-stimulating factor (G-CSF) into the circulation, sensitizing neutrophils to NETosis; the resulting accumulation of NETs in turn promotes tumor growth (94). NETs can further engage the CCDC25 receptor on tumor cells via their DNA, activating the integrin-linked kinase (ILK)–β-parvin pathway and promoting tumor cell motility and metastatic dissemination (95).

EV-induced NETosis comprises two distinct subtypes. Suicidal NETosis primarily involves NADPH oxidase activation, nuclear envelope rupture, and cell death, and typically proceeds over 3–4 hours; vital NETosis is more rapid, completing within minutes, and preserves neutrophil viability and phagocytic capacity (71). The two subtypes are activated differently: vital NETosis proceeds through the Ca²^+^/PAD4 axis, whereas suicidal NETosis proceeds through the reactive oxygen species (ROS) pathway; subtype selection is jointly determined by the biochemical composition and signaling cargo of the EVs (96).

### 4.2 Reciprocal regulation of EV secretion by NET components

Active substances released by NETs can stimulate multiple cell types to secrete large amounts of pro-inflammatory EVs, and the accumulation of such inflammatory mediators accelerates the formation of a reverse NETs → EVs positive feedback loop. Although this pathway is important, direct evidence for this reverse branch in obesity/T2DM remains limited; hypothetical inferences regarding this mechanism can therefore be drawn from related studies in autoimmune and sepsis models. In addition, disease-specific evidence in metabolic disorders is further summarized in §4.5.

Oxidized mitochondrial DNA (Ox-mtDNA) released from NETs activates the stimulator of interferon genes (STING) pathway. The pathway induces type I interferon (IFN) production. This plays a pathogenic role in systemic lupus erythematosus (SLE) and other autoimmune conditions (97). Cell-free DNA derived from NETs also synergizes with platelets to activate Toll-like receptor (TLR) signaling. The result is intravascular coagulation during sepsis and accelerated multi-organ dysfunction (87).

Activated platelets present HMGB1 to neutrophils. Autophagy is induced and NET extrusion is promoted. A platelet–NET positive feedback loop is established (87). Extracellular histones, including CitH3, act as potent independent mediators of death in sepsis. Neutralization of extracellular histones significantly improves survival in animal models (98).

Cholesterol accumulation in macrophages activates the NLRP3 inflammasome. IL-1β is secreted. Abundant NET formation in atherosclerotic plaques follows. IL-1β neutralizing antibody substantially suppresses this process (99). Activated caspase-1 cleaves gasdermin D (GSDMD). The N-terminal fragment forms pores in the plasma membrane. Non-canonical secretion of IL-1β follows, and NLRP3 activation is further amplified (100).

NE within NETs mediates DNA-dependent proteolysis of HMGB1. The C-terminal D/E repeat region of HMGB1 is removed. HMGB1 binding affinity for the TLR4–MD-2 complex is markedly enhanced. Extracellular DAMP signaling is amplified (101). NET–microparticle (MP) complexes activate TLR2/TLR4 via MP-surface HMGB1. Neutrophil adhesion and transmigration are triggered. NET formation, MP release, and neutrophil activation are coupled into a self-sustaining local positive feedback circuit (101).

### 4.3 Key Signaling pathways in the bidirectional pro-inflammatory loop

#### 4.3.1 Toll-like receptor signaling

The TLR family is the central pattern-recognition system linking EVs and NETs in a bidirectional regulatory circuit. HMGB1, oxidized lipids, and nucleic acid fragments carried by EVs engage TLR2/TLR4 at the cell surface and endosomal TLR9. MyD88-dependent recruitment of IRAK1/4 and TRAF6 follows. IκBα is then phosphorylated and degraded by the proteasome. The p65/p50 NF-κB heterodimer is released into the nucleus. Transcription of pro-inflammatory cytokines (TNF-α, IL-1β, IL-6), chemokines (CXCL8), and NADPH oxidase subunits is upregulated (102).

Oxidized mitochondrial DNA released by NETs can activate the STING pathway and induce type I IFN production, thereby lowering the NETosis threshold of neutrophils via this reverse branch (103). Rab27a GTPase coordinates the directed transport of multivesicular bodies (MVBs) to the plasma membrane and constitutes a key node regulating exosome secretion; under inflammatory conditions, accelerated exosome release substantially amplifies the EV–NET feedback loop (104).

#### 4.3.2 NLRP3 inflammasome activation

Activation of the NLRP3 inflammasome is governed by two sequential signals: the first is the priming signal, mediated by the TLR/NF-κB axis, which upregulates the expression of NLRP3 and pro-IL-1β; the second is the activation signal, such as potassium efflux or lysosomal membrane permeabilization, which drives NLRP3 oligomerization, ASC recruitment, and caspase-1 activation (105). Activated caspase-1 cleaves GSDMD to initiate pyroptosis; the N-terminal fragment of GSDMD forms membrane pores that mediate the non-canonical secretion of IL-1β and IL-18, further amplifying NLRP3 pathway activity (100). During the pathogenesis of atherosclerosis (AS), accumulation of cholesterol within macrophages can activate NLRP3 and promote IL-1β secretion; within AS plaques, the secreted IL-1β drives NLRP3 activation in neutrophils and results in extensive NET formation. Therefore, downregulation of NLRP3 can directly interrupt this feedforward loop (99).

EV-delivered endotoxin that enters the cytoplasm activates the non-canonical inflammatory caspase pathway. This pathway operates through caspase-11 in mice and through the orthologous caspase-4 and caspase-5 in humans (the manuscript previously used the combined notation “caspase-4/5/11”; the species attribution is now disambiguated). GSDMD-mediated pyroptosis follows. Large-scale inflammatory mediator release ensues (100, 105). HOCl produced by MPO oxidizes mitochondria. mtDNA is released into the cytosol. The cGAS–STING pathway is activated. Type I IFN production is enhanced. The result is positive feedback activation of neutrophils (106).

#### 4.3.3 NF-κB signaling pathway

The canonical NF-κB pathway is centered on the IκB kinase (IKK) complex. Phosphorylation-dependent degradation of IκBα releases p65/p50 into the nucleus. Transcription of hundreds of inflammation-associated genes follows. These include pro-inflammatory cytokines (IL-1β, IL-6, TNF-α), chemokines (CXCL8, CCL2), and adhesion molecules (ICAM-1, VCAM-1). NF-κB is therefore the central transcriptional integrator of inflammatory signals (107).

Extracellular HMGB1, including HMGB1 delivered by EVs, activates NF-κB via TLR4. NET formation is then induced. The HMGB1–TLR4–NF-κB axis is a critical pathway for DAMP-driven inflammatory amplification (108). NF-κB-driven IL-1β production further activates the IL-1R → MyD88 → NF-κB axis in an autocrine manner. A self-reinforcing transcriptional positive feedback loop emerges (107).

#### 4.3.4 Additional signaling pathways

##### 4.3.4.1 PAD4/CitH3 axis

PAD4 is activated by elevated intracellular Ca²^+^ and translocates to the nucleus. There it catalyzes citrullination of arginine residues on histone H3. Marked chromatin decondensation follows. PAD4 is a central regulatory node in NETosis (71).

##### 4.3.4.2 cGAS–STING pathway

Double-stranded DNA (dsDNA) released by NETs is recognized by the cytosolic DNA sensor cGAS. cGAS catalyzes synthesis of cyclic GMP-AMP (cGAMP). cGAMP activates STING → TBK1 → IRF3 signaling. Type I IFN production is induced. NF-κB is simultaneously engaged. The NETosis threshold in bystander neutrophils is lowered (109).

##### 4.3.4.3 MAPK/ERK pathway

Growth factors carried by EVs activate the Ras → Raf → MEK → ERK1/2 cascade via receptor tyrosine kinases. ERK1/2 cooperates with the AP-1 transcription factor. AP-1 synergizes with NF-κB to upregulate pro-inflammatory gene expression. ERK1/2 also phosphorylates p47phox, which facilitates NADPH oxidase assembly and accelerates NETosis (71).

### 4.4 Biological significance and regulation of the EV–NET positive feedback cascade

EVs and NETs construct a self-amplifying positive feedback cascade of sterile inflammation through extensive cross-talk among the pathways above. An initial trigger releases EVs. The EVs activate neutrophils to form NETs. NET-derived DAMPs in turn stimulate further EV secretion. The cycle repeats with escalating amplitude (71, 87, 107).

The circuit serves an adaptive function during acute infection. It enables rapid pathogen clearance. The circuit becomes pathological when dysregulated. Examples of dysregulation include impaired DNase I activity and defective EV clearance. Persistent EV–NET positive feedback then drives systemic inflammation, vascular endothelial injury, disseminated intravascular coagulation (DIC), and multi-organ dysfunction syndrome (MODS). The pathological mechanism has been substantiated in diverse diseases including sepsis, SLE, acute respiratory distress syndrome (ARDS), severe COVID-19, and atherosclerosis (93, 97, 104).

Key therapeutic strategies targeting this circuit fall into four categories. PAD4 inhibitors block CitH3 formation. NET release is suppressed. CitH3-mediated reverse stimulation of platelets and endothelial cells is also reduced (71). DNase I directly degrades extracellular DNA. It disassembles NETs and attenuates STING pathway activation. The positive feedback loop is interrupted. NLRP3 inhibitors (e.g., MCC950) concurrently block EV-induced NET formation and NET-driven excessive pro-inflammatory EV secretion (104). Antagonism of the HMGB1–TLR2/TLR4 axis disrupts NET–MP complex-triggered neutrophil recruitment. Inflammatory cascade amplification is effectively suppressed (101).

**Figure 3:**
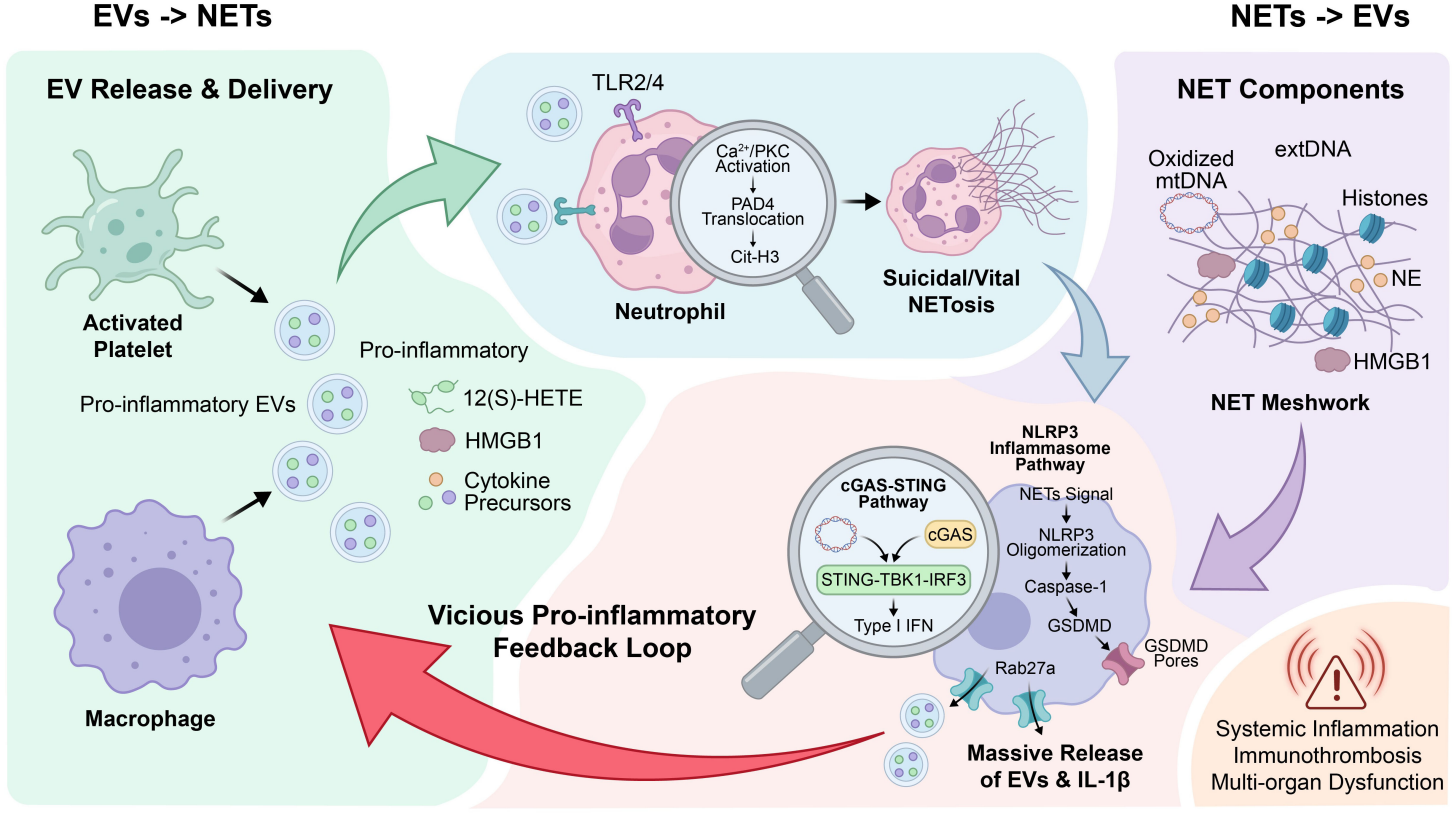
The bidirectional pro-inflammatory feedback loop between extracellular vesicles (EVs) and neutrophil extracellular traps (NETs). A self-reinforcing inflammatory circuit is established by reciprocal crosstalk between EVs and NETs. The circuit is organized as a left–right symmetric closed feedback loop. EVs → NETs (left/upper arc): In response to initial inflammatory cues, source cells release pro-inflammatory EVs enriched in DAMPs. Source cells include activated platelets, macrophages, and endothelial cells. The DAMP cargo includes HMGB1, 12(S)-HETE, and cytokine precursors. These EVs engage neutrophil TLR2/4. Ca²^+^/PKC signaling, PAD4 nuclear translocation, and histone citrullination (Cit-H3) follow. Suicidal and vital NETosis are driven. NETs → EVs (right/lower arc): The extruded NET meshwork exposes a second wave of DAMPs. The new DAMPs include oxidized mitochondrial DNA (mtDNA), extracellular DNA (extDNA), histones, NE, and HMGB1. These DAMPs activate surrounding macrophages through two pathways. The first is the cGAS–STING–TBK1–IRF3 axis. The pathway drives Type I IFN production. The second is the NLRP3 inflammasome–caspase-1–GSDMD pathway. (Note: the non-canonical inflammasome pathway, not explicitly depicted, operates via caspase-11 in mice and via the orthologous caspase-4 and caspase-5 in humans; see §4.3.2 of the main text.) GSDMD pore formation, in concert with Rab27a-mediated vesicular trafficking, then drives massive secondary release of newly formed pro-inflammatory EVs and IL-1β. The new EVs feed back to reinitiate NETosis. If unresolved, the vicious EV–NET cascade amplifies local and systemic sterile inflammation, immunothrombosis, and multi-organ dysfunction. Note that the precise wiring of the reciprocal NETs → EVs arm in obesity/T2DM has been inferred predominantly from sepsis, COVID-19, and autoimmune models. Direct demonstration in metabolic disease remains an active research priority (see §4.5). Schematic produced with BioRender.com (publication license obtained by the corresponding author). cGAS, cyclic GMP–AMP synthase; Cit-H3, citrullinated histone H3; DAMPs, damage-associated molecular patterns; EV, extracellular vesicle; extDNA, extracellular DNA; GSDMD, gasdermin D; HMGB1, high-mobility group box 1; 12(S)-HETE, 12(S)-hydroxyeicosatetraenoic acid; IFN, interferon; IRF3, interferon regulatory factor 3; mtDNA, mitochondrial DNA; NE, neutrophil elastase; NET, neutrophil extracellular trap; NLRP3, NOD-like receptor family pyrin domain–containing 3; PAD4, peptidylarginine deiminase 4; PKC, protein kinase C; STING, stimulator of interferon genes; TBK1, TANK-binding kinase 1; TLR, Toll-like receptor.

The EV–NET bidirectional pro-inflammatory loop is a highly integrated molecular network in inflammatory pathophysiology. **Figure 3** illustrates the mechanism of cyclic amplification of the EV–NET bidirectional pro-inflammatory loop *in vivo*.

### 4.5 Evidence calibration: direct vs inferred mechanisms in metabolic disease (new subsection — reviewer comment C1)

Much of the mechanistic detail of the EV–NET feedback circuit summarized in §§4.1–4.4 has been directly demonstrated in non-metabolic settings. These settings include sepsis (87, 90), severe COVID-19 (93), systemic lupus erythematosus (97), and cancer (94, 95). To avoid over-extrapolation, we distinguish here between steps directly demonstrated in obesity/T2DM and steps inferred by analogy.

#### 4.5.1 Steps directly demonstrated in obesity/T2DM

Adipose- and ATM-derived EVs carrying miR-155 and miR-27a induce insulin resistance and impair β-cell function in mouse and human-tissue models (9, 56, 110). Circulating NET biomarkers (MPO–DNA, CitH3) are elevated in obese and diabetic patients. Their levels correlate with HOMA-IR, BMI, and HbA1c (15, 16). PAD4 deletion or pharmacological inhibition reduces diabetic retinopathy, nephropathy, and cardiomyopathy in rodent models (111–113). DNase I-mediated NET clearance promotes atherosclerosis regression in diabetic mice (17). Rosi-ATM-sEVs carrying miR-690 reverse obesity-induced insulin resistance *in vivo* (58).

#### 4.5.2 Steps inferred by analogy and still awaiting direct demonstration in metabolic disease

The precise molecular wiring of the *reciprocal EV → NET → EV amplification loop* in obesity has been documented primarily in sepsis, autoimmune, and tumor models (93, 97, 104). Three specific steps remain to be confirmed in metabolic disease. First is the role of the cGAS–STING axis in NET-driven secondary EV release from adipose-tissue or hepatic macrophages. Second is GSDMD-pore-mediated non-canonical EV release downstream of NLRP3 activation in this context. Third is Rab27a-dependent secondary EV trafficking driven by NET-derived DAMPs. Whether the temporal kinetics and quantitative thresholds of these steps in chronic metabolic inflammation match those characterized in acute settings remains an open question. The question is experimentally addressable.

#### 4.5.3 Translational implication

This calibration matters for therapeutic prioritization. Agents whose primary mechanism (PAD4 inhibition, DNase I, NLRP3 blockade) has direct metabolic-disease evidence warrant near-term clinical evaluation in obesity/T2DM. Agents predicated chiefly on the inferred reciprocal arm (cGAS–STING antagonists, Rab27a-pathway modulators) require interim mechanistic validation in metabolic models before clinical investment. We have flagged inferred steps explicitly throughout §§4 and 5.

## 5 Role of the sterile inflammatory cascade in obesity-induced T2DM and its multi-organ complications

### 5.1 Activation of the EV–NET axis in the obese metabolic microenvironment and the formation of sterile inflammation

Chronic low-grade inflammation induced by obesity is a core pathological feature of T2DM, driven by a series of endogenous danger signals and manifesting as “sterile inflammation,” which does not depend on infection for its formation. Under obese conditions, visceral adipose tissue (VAT) continuously expands and undergoes cell death; these hypertrophic and necrotic adipocytes persistently release DAMPs, such as free fatty acids (FFAs), uric acid crystals, and oxidized phospholipids, and further induce adipose tissue macrophages (ATMs) to shift from the anti-inflammatory M2 phenotype to the pro-inflammatory M1 phenotype, secreting large amounts of TNF-α, IL-1β, and IL-6 (83).

Adipocyte-derived extracellular vesicles (AdEVs) play a key role in amplifying sterile inflammation. In the obese environment, EVs derived from M1-type ATMs are enriched in miR-155. miR-155 can activate the downstream NF-κB signaling pathway by inhibiting Socs1 (Suppressor of Cytokine Signaling 1), further promoting M1 polarization of macrophages and cascade-amplifying the release of inflammatory cytokines (53). miR-155 also acts on Pparg in adipocytes, hepatocytes, and skeletal muscle cells, being transferred unidirectionally from ATMs to recipient parenchymal cells, thereby accelerating systemic insulin signaling impairment. AdEVs derived from hypertrophic adipocytes are enriched in miR-27a, which can suppress PPARγ-mediated glucose uptake (108). In addition, adipose-tissue-derived EVs activate recipient cells via the TLR4/NF-κB pathway, sustaining activation of the pro-inflammatory loop (112).

The high-fat, high-glucose microenvironment is a potent trigger of NET formation. PAD4 catalyzes citrullination of histone H3 (CitH3), which in turn causes chromatin decondensation, and can also synergize with ROS produced by NADPH oxidase to drive NETosis (68). In obese individuals, circulating NET biomarkers (MPO–DNA complexes, CitH3) are significantly elevated, and the degree of elevation is independently positively correlated with BMI and HOMA-IR (16). NET infiltration can be detected in the adipose tissue of both obese humans and high-fat diet (HFD)-fed mice, and the timing of infiltration is consistent with the trend of local neutrophil accumulation and elevation of related inflammatory markers (15).

The NLRP3 inflammasome is a central molecular hub of sterile inflammation. Under sterile conditions, PAD4 upregulates the protein levels of NLRP3 and ASC in neutrophils, promoting NLRP3 inflammasome assembly. Knockout of the NLRP3 gene substantially weakens NET formation, indicating that PAD4 and NLRP3 act in a mutually reinforcing manner (113). Oxidized lipids carried by EVs, upon internalization by cells, activate NLRP3 through lysosomal damage and amplify adipose tissue inflammatory injury via the NLRP3 pathway (102). Hypochlorous acid (HOCl) generated by MPO within NETs oxidizes mitochondria, leading to cytosolic mtDNA release and activation of the cGAS–STING pathway, which synergizes with NLRP3 to further amplify inflammatory signaling within the tissue (101).

### 5.2 Molecular mechanisms by which EV–NET interactions accelerate insulin resistance

Insulin resistance (IR) is an important pathological transition in the progression from obesity to T2DM. As mentioned above, small EVs (Rosi-ATM-sEVs) released by adipose tissue macrophages from rosiglitazone-treated obese mice are enriched in miR-690, which can improve insulin sensitivity in recipient adipocytes, myotubes, and hepatocytes by inhibiting Nadk. On the other hand, although the donor macrophages exhibit certain features of the M2 phenotype, according to published studies these ATMs are TZD-induced ATMs rather than classical IL-4-driven M2 macrophages (54, 55). In contrast, exosomes derived from obese M1-type ATMs carry miR-155, which exerts the opposite effect via PPARγ. The above results show that the metabolic regulatory features of ATM-derived exosomes exhibit specific bidirectional effects depending on ATM subtype (54). In addition, miR-27a carried by AdEVs can downregulate PPARγ expression in skeletal muscle and hepatocytes, suppress GLUT4 membrane translocation, and reduce glucose uptake (108). The ceramide carried by these vesicles activates protein phosphatase 2A (PP2A), mediating Akt dephosphorylation and directly disrupting a key node of downstream insulin signaling (114).

Notably, not all obesity-related EV cargoes are pathogenic. Hepatocyte-derived exosomes from early-onset obese mice are enriched in miR-3075, which can activate the FAK–IRS-1 axis in skeletal muscle and improve insulin sensitivity (10). This finding runs counter to the mainstream view that “obesity EVs are uniformly pathogenic” and provides new translational evidence for EVs in the prevention and treatment of obese T2DM. Further studies have shown that along the time course of obesity progression, EV cargo composition is dynamically changing (early-onset vs. chronic), and there are also marked differences among cells of different origins (hepatocytes vs. adipocytes vs. ATMs). Detailed analysis of the circulating EV pool in obesity reveals that the EVs therein contain both pathogenic and compensatory protective subpopulations. Therefore, the therapeutic challenge for obese T2DM should not be merely the comprehensive suppression of EV secretion, but rather how to efficiently suppress the secretion and assembly of pathogenic subpopulations while maximally preserving protective subpopulations. This perspective will be further elaborated in §6.4 and §6.7.

Activation of the NLRP3 inflammasome is one of the key factors in IR progression. The IL-1β it secretes can promote serine phosphorylation of IRS-1 via JNK while simultaneously activating the IKKβ–NF-κB axis, and the superposition of the two directly impairs insulin signaling in the liver, skeletal muscle, and adipose tissue (115). NET-derived DNA–histone complexes activate the TLR9 pathway in Kupffer cells, triggering NF-κB-dependent hepatic inflammation, aggravating hepatic IR, and further increasing hepatic glucose output (116). Obesity-associated hyperlipidemia continuously stimulates neutrophils to produce NETs via TLR4 signaling, and these NETs in turn induce secretion of platelet-derived EVs (PEVs), ultimately forming a vicious cycle of FFA → NETs → PEVs → systemic inflammation → IR (94). In this process, visceral AdEVs can exhibit a more pronounced pro-inflammatory miRNA signature, and their ability to accelerate IR even exceeds that of subcutaneous adipose-derived EVs (80).

### 5.3 The EV–NET positive feedback mechanism in pancreatic β-cell injury

Islet amyloid polypeptide (IAPP) oligomers can directly activate the NLRP3 inflammasome in β cells through lysosomal membrane permeabilization and upregulation of cathepsin B, triggering downstream activation of caspase-1 and release of IL-1β. This process is one of the initiating points of islet inflammation in T2DM (118). Obese ATMs release EVs enriched in miR-155; after these EVs are internalized by pancreatic β cells, the glucose-stimulated insulin secretion pathway is suppressed, and these EVs also promote abnormal β-cell proliferation via the miR-155–Mafb axis (9). On the other hand, advanced glycation end products (AGEs) can activate the islet NLRP3 inflammasome via the TXNIP–NLRP3 pathway, directly damaging β-cell ultrastructure; knockout of the NLRP3 gene significantly improves the morphological and functional integrity of islets under AGE intervention (119).

Under diabetic conditions, marked upregulation of PAD4 in neutrophils exacerbates NETosis, and CitH3 and free histones in turn activate NF-κB in β cells via TLRs, causing iNOS overexpression; the excessive nitric oxide (NO) directly disrupts mitochondrial oxidative phosphorylation, ultimately accelerating β-cell apoptosis (120). This indicates that the PAD4–NETosis axis is a key pathway in β-cell injury, and in PAD4-deficient mice, islet NET deposition is significantly reduced and the β-cell apoptosis rate is substantially decreased (121). On the other hand, the chronic hyperglycemic environment of T2DM also persistently activates the AGEs–RAGE axis in islet endothelial cells, accelerating β-cell apoptosis by maintaining the EV–NET positive feedback loop (124).

β cells themselves also actively release EVs during the injury process; these EVs are often enriched in HSP70 and mtDNA, which can activate adjacent islet macrophages and trigger further NET release, ultimately forming a self-amplifying cycle of β-cell injury → β-EVs → NETs → further β-cell injury (122). Pyroptotic bodies generated by GSDMD-mediated pyroptosis are enriched in HMGB1, and HMGB1, as a potent DAMP, can amplify the local inflammatory microenvironment in islets (98); therefore, inhibition of the NLRP3–TXNIP axis under hypoxic conditions can significantly improve islet survival and post-transplant functional recovery (123).

### 5.4 The gut–adipose–NET axis in obesity-associated diabetes and its complications

Mounting evidence confirms that the gut is a major source of systemic NETosis in obesity. A high-fat diet causes intestinal barrier dysfunction, allowing bacterial LPS, peptidoglycan, and bile acid-derived signals to translocate into the portal and systemic circulation—a phenomenon known as metabolic endotoxemia (114). Translocated LPS binds to TLR4/NLRP3 on adipose tissue macrophages and circulating neutrophils, substantially lowering the NETosis threshold and ultimately amplifying adipose tissue inflammatory effects.

#### 5.4.1 Direct experimental evidence in T2DM

In fact, intestinal NET deposition itself damages the intestinal epithelial barrier (111). Via the pathway “intestinal NETs → barrier disruption → LPS translocation → systemic inflammation,” it directly leads to the chronic low-grade inflammatory environment of T2DM. In db/db mice, daily intraperitoneal injection of the PAD4 inhibitor GSK484 (4 mg/kg/d) produces multiple effects: intestinal NET density decreases, barrier integrity is restored, the abundance of immature neutrophils in circulation drops substantially, bone marrow neutrophil retention increases, and these combined factors substantially slow the progression of diabetic retinopathy (111). Clinical studies corroborate these results—an analysis of 58 T2DM patients with retinopathy showed that neutrophilia was significantly associated with elevated markers of intestinal permeability. This finding, from the NET perspective, confirms the existence of a gut–retina axis in human diabetic microvascular disease (111).

#### 5.4.2 Broader implications for multi-organ pathology

Based on this gut-centered framework, the EV–NET axis can be redefined: intestinal NETs and gut-derived EVs (from intestinal epithelial cells and the microbiota) jointly feed into the same circulating EV–NET pool. This pool drives the adipose, hepatic, islet, and microvascular pathologies described in §§5.2, 5.3, 5.5, and 5.6. Conversely, the gut–liver axis in NASH (§5.6), the gut–retina axis in diabetic retinopathy (111), and the gut–kidney axis in diabetic kidney disease can all be regarded as anatomically distinct downstream manifestations of the same upstream barrier failure. Recent NAFLD studies further indicate that microbiota-modified bile acids and short-chain fatty acid imbalance are important regulators of neutrophil priming and represent potential key intervention nodes (115).

#### 5.4.3 Therapeutic relevance

Given the importance of the gut EV–NET axis, its targeted intervention is as important as systemic anti-NET strategies. Candidate interventions include: dietary modulation (high-fiber, Mediterranean-style diet); prebiotics and specific probiotics; GLP-1 receptor agonists, which independently enhance barrier function; and local intestinal delivery of DNase I or PAD4 inhibitors. Notably, gut-restricted delivery may achieve barrier protection while preserving systemic innate immune capacity. We comprehensively discuss the related safety considerations in §6.7.

### 5.5 EV–NET mechanisms in vascular endothelial dysfunction and diabetic vascular complications

Hyperglycemia stimulates endothelial cells to secrete endothelial-derived EVs (EEVs) enriched in tissue factor (TF), ICAM-1, and P-selectin. After entering the circulation, these EEVs activate neutrophils and trigger NET formation (34). CitH3 within NETs in turn activates endothelial NF-κB, upregulates adhesion molecules, promotes transendothelial monocyte migration, and accelerates atherosclerotic plaque formation; free NE degrades the endothelial glycocalyx, and MPO-derived HOCl causes eNOS uncoupling, further aggravating endothelial dysfunction (67).

The PAD4–NET axis is an important therapeutic target in diabetic kidney disease (DKD): NETs activate the glomerular NLRP3 inflammasome, disrupt the glomerular endothelial barrier, and ultimately cause proteinuria; the PAD4 inhibitor GSK484 significantly ameliorates NET-induced glomerular endothelial dysfunction (76). In macrovascular complications, NETs persist within diabetic atherosclerotic plaques, leading to sustained activation of macrophage NLRP3 and glycolytic pathways and suppressing downstream inflammation resolution. Clearance of intimal NETs with DNase I significantly attenuates macrophage inflammation and promotes atherosclerosis regression (17). Diabetes activates platelets to secrete large amounts of PEVs; once these PEVs recruit neutrophils, they induce NETs to serve as scaffolds for coagulation factors, ultimately promoting intravascular immunothrombosis (96). Finally, dysfunctional mitochondria continuously release mtDNA, which sustains chronic vascular inflammation by activating the cGAS–STING–NF-κB axis in vascular cells (106).

### 5.6 Multi-organ molecular evidence: liver, adipose tissue, and pancreas

We previously discussed the pro-inflammatory features of EVs from multiple organs; here we further elaborate on these features.

#### 5.6.1 Liver

EVs produced by lipotoxic hepatocytes show markedly elevated miR-192-5p, which activates Kupffer cells via a Rictor-dependent pathway and amplifies local inflammation during NASH progression (116). Concurrently, NASH-induced neutrophil-derived NETs (regulated by S1PR2) promote hepatic fibrogenesis via the Rho/ROCK pathway (117), and this process is further amplified by pyroptosis: hepatic macrophages, after engulfing NETs, undergo pyroptosis via the AIM2 inflammasome (118). Clinically, circulating NET biomarkers are significantly elevated in NASH patients and are often positively correlated with the NAFLD activity score and fibrosis stage (119).

#### 5.6.2 Adipose tissue

In the visceral adipose tissue (VAT) of obese patients with type 2 diabetes, neutrophil NET infiltration is clearly observable, with CitH3 and NET-DNA complexes co-localized at the central region of crown-like structures (CLSs). Given that CLSs themselves represent clearance microenvironments formed by macrophages surrounding dead adipocytes, NET deposition at this site can significantly block adipocyte clearance and, on this basis, further amplify local inflammatory signaling (119, 120). Visceral-derived AdEVs are enriched in pro-inflammatory miRNAs and have a stronger capacity to induce insulin resistance (IR) than subcutaneous adipose-derived EVs (83). On this basis, engineered EVs delivering anti-inflammatory miRNAs can effectively ameliorate obesity-associated metabolic dysfunction in mice (121).

#### 5.6.3 Pancreas

In the pancreatic microcirculation, NET deposition impairs local blood flow and oxygen supply and further induces activation and fibrogenic processes in pancreatic stellate cells (122). Integrating EV-mediated long-range signaling with NET-mediated local tissue damage, the combined effects may collectively accelerate the progressive decline of islet function.

### 5.7 Therapeutic implications of targeting the EV–NET axis

A systematic synthesis of the pathophysiological processes described above identifies four pharmacologically actionable intervention nodes: (i) PAD4 inhibition (e.g., GSK484), which blocks the rate-limiting step of NETosis while attenuating NLRP3 activation (102, 123); (ii) DNase I-mediated NET degradation, which clears formed NETs and indirectly suppresses downstream EV release (17); (iii) MCC950 or related NLRP3-inhibiting agents, which interrupt the EV → NET → EV reciprocal loop at the central inflammasome node (124); and (iv) clinically deployed antidiabetic drugs with documented anti-NET or anti-inflammasome activity, such as the widely used metformin and SGLT2 inhibitors (123, 125). In §6, we evaluate each of these in detail and explicitly specify the level of available clinical evidence.

**Figure 4:**
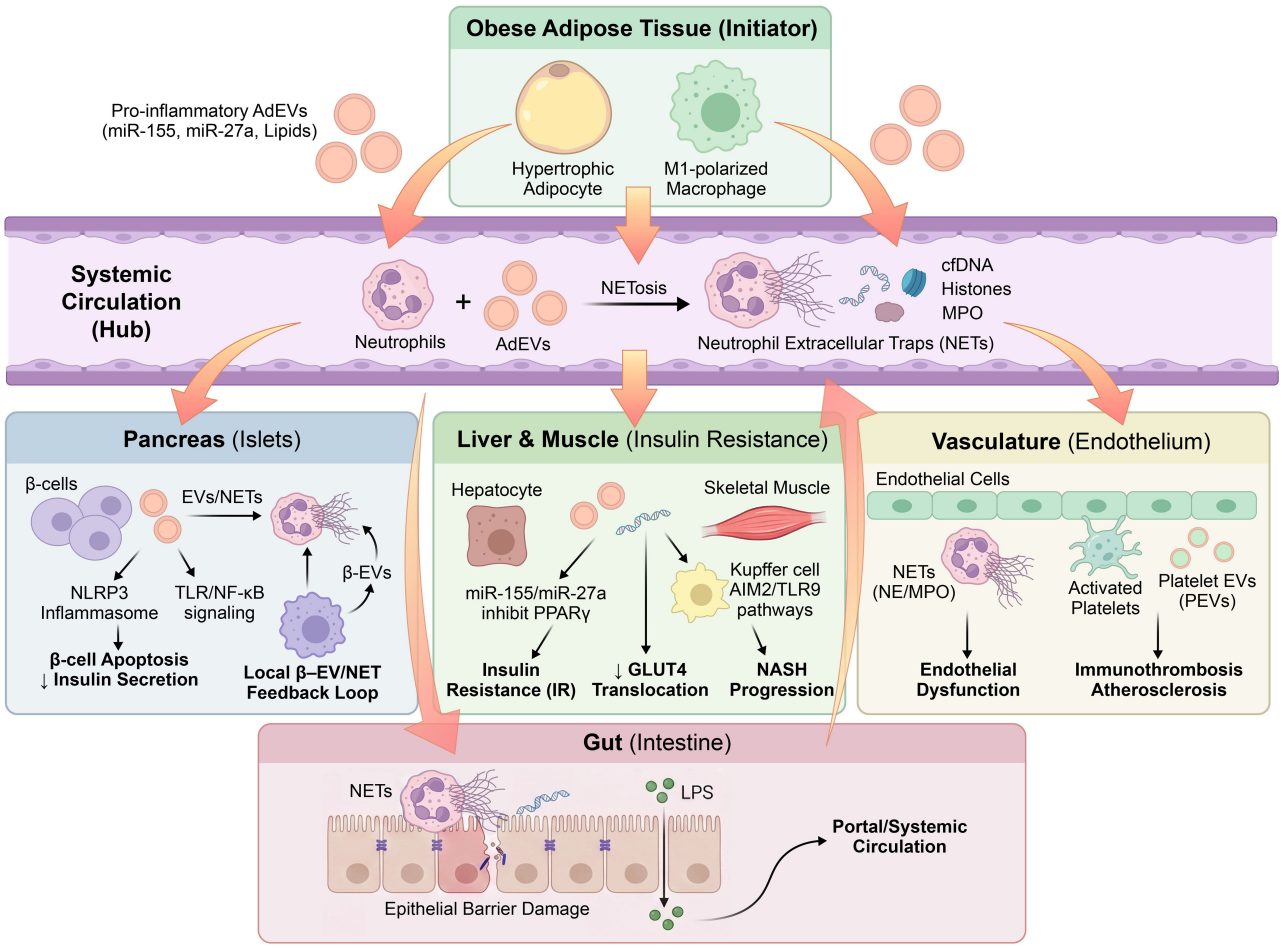
The EV–NET-driven sterile inflammation cascade and multi-organ dysfunction in obesity-induced type 2 diabetes. Initiator (top). In obesity, hypertrophic adipocytes and M1-polarized ATMs release pro-inflammatory adipose-derived EVs (AdEVs). The AdEVs are enriched in DAMPs and pathogenic miRNAs (miR-155, miR-27a) together with lipotoxic lipids. Hub (middle, systemic circulation). Circulating AdEVs converge with neutrophils to trigger PAD4- and ROS-dependent NETosis. NETs decorated with cfDNA, histones, NE, and MPO are released. This systemic hub disseminates sterile inflammation to four principal target organ systems (bottom). (1) Pancreatic islets (left): Infiltrating EVs and NETs activate the NLRP3 inflammasome and TLR/NF-κB signaling in β-cells. β-cell apoptosis follows. Insulin secretion is reduced. A local β-cell-derived EV (β-EV)/NET feedback loop further reinforces the process. (2) Liver and skeletal muscle (middle): AdEV-delivered miR-155/miR-27a suppress PPARγ and impair GLUT4 membrane translocation. NETs activate Kupffer cell AIM2/TLR9 signaling. Together these drive systemic insulin resistance and NASH progression. (3) Vasculature (right): NET-derived NE/MPO disrupt endothelial barrier function. Activated platelet and platelet EV (PEV) release is promoted. Immunothrombosis and atherosclerosis follow. (4) Gut (newly added panel). Intestinal NETs damage the epithelial barrier. Portal and systemic translocation of LPS increases. The gut feeds back to the systemic EV–NET hub. This mechanism is directly demonstrated in db/db mice and in 58 T2DM patients with retinopathy (111). The gut–adipose–retina axis is detailed in §5.4 of the main text. For therapeutic strategies targeting this multi-organ vicious cycle, see **Figure 5**. The “Candidate intervention nodes/Therapeutic Targets” inset previously included in this figure has been removed in the revised manuscript to avoid duplication with **Figure 5** (per Reviewer Comment E2). Schematic produced with BioRender.com (publication license obtained by the corresponding author). AdEVs, adipose-derived EVs; AIM2, absent in melanoma 2; ATM, adipose tissue macrophage; β-EVs, β-cell-derived EVs; cfDNA, cell-free DNA; EV, extracellular vesicle; GLUT4, glucose transporter type 4; IR, insulin resistance; LPS, lipopolysaccharide; MPO, myeloperoxidase; NASH, non-alcoholic steatohepatitis; NE, neutrophil elastase; NET, neutrophil extracellular trap; NF-κB, nuclear factor-κB; NLRP3, NOD-like receptor family pyrin domain–containing 3; PAD4, peptidylarginine deiminase 4; PEVs, platelet-derived EVs; PPARγ, peroxisome proliferator-activated receptor gamma; ROS, reactive oxygen species; TLR, Toll-like receptor.

**Figure 5:**
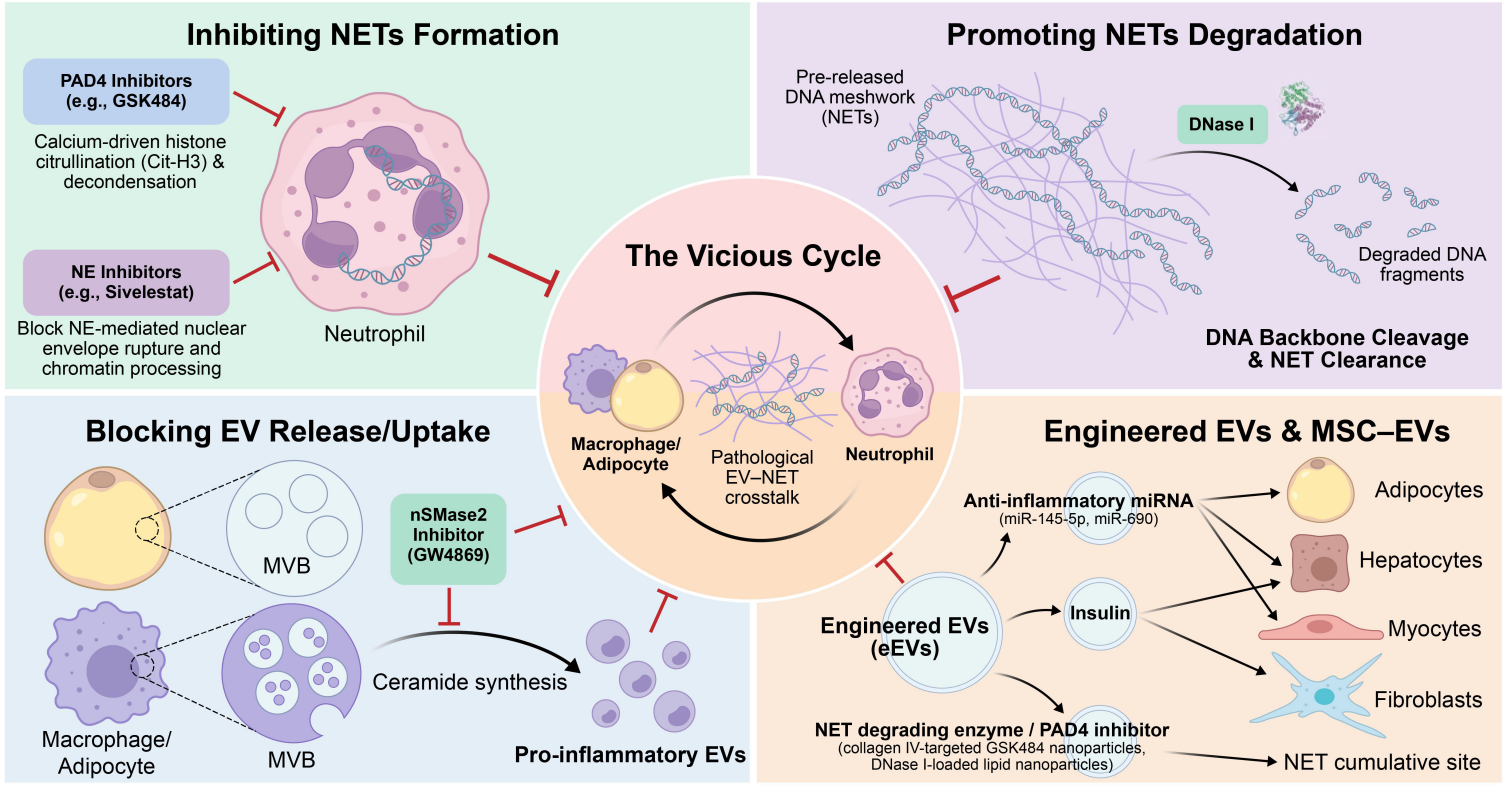
Therapeutic strategies targeting the extracellular vesicle (EV)–neutrophil extracellular trap (NET) pathological axis in obesity-related diabetes. The central panel depicts the self-reinforcing vicious cycle between adipose tissue macrophages/adipocytes and neutrophils. Pathological EV–NET crosstalk in this cycle amplifies chronic inflammation, insulin resistance, and multi-organ injury. Five complementary therapeutic strategies are designed to disrupt the loop. **(A)** Inhibiting NET formation (top-left, green panel). PAD4 inhibitors, exemplified by GSK484, block calcium-dependent citrullination of histone H3 (Cit-H3). Chromatin decondensation, the rate-limiting step of NETosis, is therefore prevented. NE inhibitors such as sivelestat suppress NE-mediated nuclear envelope rupture and chromatin processing. NET extrusion is thereby prevented. **(B)** Promoting NET degradation (top-right, purple panel). DNase I hydrolyzes the DNA backbone of pre-released NET meshwork. Degraded DNA fragments are generated. NETs are cleared from the inflammatory microenvironment. NET-driven amplification of EV biogenesis is interrupted. **(C)** Blocking EV release and uptake (bottom-left, blue panel). The nSMase2 inhibitor GW4869 suppresses ceramide synthesis. Ceramide is required for inward budding of MVB membranes in adipocytes and macrophages. EV biogenesis is reduced. The distant propagation of insulin resistance is attenuated. **(D)** Engineered EVs and MSC-derived EVs — three *separate* strategies, not a single multi-cargo platform (bottom-right, orange panel). Three independent lines of investigation are illustrated. Each is distinguished by the cargo loaded into the EV scaffold: (D1) eEVs loaded with anti-inflammatory miRNAs. Examples include miR-145-5p and miR-690 enriched in Rosi-ATM-sEVs. They are delivered to adipocytes, hepatocytes, myocytes, and ATMs. Insulin sensitivity is restored through *Nadk* suppression or L-selectin downregulation (58, 61). M(D2) eEVs loaded with insulin (typically by electroporation). They are delivered to hepatocytes and fibroblasts for glucose lowering. They confer absorption advantages over native insulin in selected contexts (147). (D3) eEVs or nanoparticles loaded with NET-degrading enzymes or PAD4 inhibitors. Examples include collagen IV-targeted GSK484 nanoparticles and DNase I-loaded lipid nanoparticles. They are delivered to sites of pathological NET accumulation. Site-specific NET clearance is achieved (129, 152). D1, D2, and D3 are conceptually and experimentally independent. The unified panel in earlier drafts of this figure was misleading. It implied a single multi-cargo platform. Combination loading remains an aspirational future direction. It has not been validated in obesity-related diabetes. **(E)** Repurposing existing antidiabetic agents with documented anti-EV–NET activity (new sub-panel — reviewer comment F5; see §6.6.3). Metformin inhibits PKC–NADPH oxidase. Circulating NET biomarkers are directly reduced in T2DM/prediabetes patients, independently of glycemic control (125). SGLT2 inhibitors elevate circulating β-hydroxybutyrate. β-hydroxybutyrate suppresses NLRP3 inflammasome activation. SGLT2 inhibitors have documented cardiorenal benefit in cardiovascular outcome trials (123). These two agents are the most translatable elements of the framework. Additional candidates under preliminary investigation include GLP-1 receptor agonists (gut-barrier strengthening), pioglitazone (a TZD analog of rosiglitazone), statins, and colchicine. Red blunt-ended arrows (⊣) indicate inhibitory actions of therapeutic agents on their molecular targets. Black arrows denote the direction of pathological signaling or therapeutic cargo delivery. Schematic produced with BioRender.com (publication license obtained by the corresponding author). Cit-H3, citrullinated histone H3; cfDNA, cell-free DNA; CVOT, cardiovascular outcome trial; eEVs, engineered extracellular vesicles; EVs, extracellular vesicles; GLP-1, glucagon-like peptide-1; MSC-EVs, mesenchymal stem cell-derived extracellular vesicles; MVB, multivesicular body; NE, neutrophil elastase; NETs, neutrophil extracellular traps; nSMase2, neutral sphingomyelinase 2; PAD4, peptidylarginine deiminase 4; Rosi-ATM-sEVs, small extracellular vesicles from adipose tissue macrophages of rosiglitazone-treated obese mice; SGLT2i, sodium–glucose cotransporter 2 inhibitor; TZD, thiazolidinedione.

As shown in **Figure 4**, the sterile inflammation driven by the EV–NET axis transforms obesity-associated metabolic stress into continuously amplified systemic inflammatory signals; this transformation is primarily achieved through multi-organ positive feedback networks involving adipose tissue, gut, liver, pancreas, and the vasculature, thereby accelerating T2DM onset and complication progression. In the subsequent **Figure 5**, we further dissect the spatiotemporal regulatory dynamics of the EV–NET–NLRP3–cGAS–STING multi-axis crosstalk network and systematically describe precision anti-inflammatory therapies and potential interventions targeting sterile inflammation in T2DM progression (102, 123).

## 6 Therapeutic strategies targeting the EV–NET axis

Extracellular vesicles (EVs) and neutrophil extracellular traps (NETs) establish a mutually reinforcing loop of inflammatory and metabolic injury in the pathological progression of obesity-related diabetes. Neutrophil-derived EVs and NETs share multiple effector molecules. Their reciprocal activation within the same microenvironment amplifies inflammatory signaling (20). EVs act as endogenous danger signals that activate pattern recognition receptors and trigger NETosis. Conversely, NET components stimulate surrounding cells to generate additional EVs. The principal NET components involved are histones, cell-free DNA (cfDNA), and neutrophil elastase. A positive feedback circuit is established (19). Disrupting this pathological axis could improve insulin resistance, β-cell dysfunction, and chronic multi-organ inflammation simultaneously. Current therapeutic strategies operate at four levels: inhibiting NET formation; promoting NET degradation; blocking the biogenesis or uptake of pathogenic EVs; and engineered EV-based drug delivery. A fifth, increasingly important translational approach is repurposing established antidiabetic agents that incidentally modulate the EV–NET axis. This approach is discussed separately in §6.6.3. An integrated overview is summarized in **Figure 5**. A horizontal comparison of these five strategy classes—including their molecular targets, organ-specific preclinical evidence, advantages, limitations, and current stage of clinical evidence—is presented in **Table 1**.

**Table 1:** Horizontal comparison of five therapeutic strategy classes targeting the EV-NET axis in obesity-related diabetes.

Strategy class	Core target or intervention	Key evidence in the manuscript	Translational note
Inhibiting NET formation	PAD4 inhibition (GSK484, PAD4 deficiency); neutrophil elastase inhibition (sivelestat, elafin); and ROS or calcium-calcineurin blockade (84, 111-113, 126-135).	GSK484 reduced intestinal NETs and slowed diabetic retinopathy in db/db mice (111). PAD4 deficiency protected against cardiac and renal injury (112). GSK484 also improved diabetic nephropathy indices (113).	Acts upstream on NETosis, but selectivity is limited and host-defense concerns remain. Evidence is still mainly preclinical, with no completed obesity-related diabetes trial.
Promoting NET degradation	DNase I-mediated NET clearance, DNase1L3-complemented degradation, and engineered dual-active DNase1 variants (17, 136-140).	DNase I promoted atherosclerosis regression and reduced macrophage inflammation in diabetic models (17). It also lowered tissue NET burden in vasculitic organ injury models and may reduce thrombotic risk (137-139).	Useful for clearing formed NETs, but limited by short half-life, incomplete clearance, and resistance in some settings. Clinical exploration exists outside obesity-related diabetes.
Blocking pathogenic EV biogenesis or uptake	nSMase2 inhibition with GW4869; selective blockade of pathogenic EV uptake or cargo neutralization (19, 20, 50, 141-144).	GW4869 blocked transfer of miR-141-3p from obese adipose EVs to hepatocytes and interrupted hepatic insulin resistance signaling (142, 143). However, glucose tolerance worsened slightly in lean DIO mice, suggesting beneficial EV pools are also affected (50).	Targets the EV arm of the loop, but broad EV suppression risks removing protective vesicles. This remains a preclinical strategy.
Engineered EV-based drug delivery	Insulin-loaded exosomes, Rosi-ATM-sEVs, MSC-derived EVs, miR-145-5p replacement, and anti-NET payload delivery platforms (21, 58, 61, 145-153).	Rosi-ATM-sEVs improved insulin sensitivity without rosiglitazone-like weight gain (58). MSC-EVs improved hepatic steatosis and insulin signaling in HFD or HFD-STZ models (149, 150). Targeted GSK484 or DNase I plus NE inhibitor delivery also reduced NET-associated injury (129, 152).	Potentially precise and biocompatible, but manufacturing, clearance, and quality control remain major barriers. Human EV trials are still exploratory and heterogeneous.
Repurposing established antidiabetic agents	Metformin, SGLT2 inhibitors, and other clinically used modulators with plausible EV-NET or NLRP3 effects (58, 123, 125).	Metformin reduced circulating NET-associated components in T2DM or prediabetes and also attenuated NETosis in vitro (125). SGLT2 inhibitors may weaken the EV-NET-NLRP3 amplification node through beta-hydroxybutyrate-linked suppression of inflammasome signaling (123).	Most realistic near-term option because safety and prescribing pathways already exist. Current support is still mechanistic or biomarker-based rather than direct proof of EV-NET-mediated clinical benefit.

EV, extracellular vesicle; NET, neutrophil extracellular trap; PAD4, peptidylarginine deiminase 4; NE, neutrophil elastase; nSMase2, neutral sphingomyelinase 2; HFD, high-fat diet; STZ, streptozotocin; T2DM, type 2 diabetes mellitus.

### 6.2 Therapeutic strategies targeting NET formation

#### 6.2.1 PAD4 inhibitors

Peptidylarginine deiminase 4 (PAD4) is the central regulatory enzyme of NETosis. PAD4 catalyzes citrullination of histone arginine residues in a calcium-dependent manner. Chromatin decondensation follows. Citrullination is the rate-limiting step in NET release (126). GSK484 is the most extensively studied selective, reversible PAD4 inhibitor. Its IC_50_ is 50 nM under calcium-free conditions (126).

In db/db mice, daily intraperitoneal injection of GSK484 (4 mg/kg/d) for four weeks produced multiple effects. Intestinal NET density was significantly reduced. Gut epithelial senescence was attenuated. The abundance of premature neutrophils in blood was decreased. Neutrophil retention in the bone marrow was promoted. Diabetic retinopathy progression was slowed (111). Parallel analysis of 58 type 2 diabetes patients with varying degrees of retinopathy revealed a significant association between neutrophilia and elevated markers of intestinal permeability. This finding supports a gut–retina axis in diabetic complications (111). In streptozotocin (STZ)-induced diabetic models, PAD4^-^/^-^ mice were protected against both cardiac and renal injury. High glucose triggered inflammasome activation in neutrophils. Wild-type diabetic mice developed increased myocardial and renal NET deposition, elevated IL-1β, and progressive fibrosis. All of these were absent in PAD4^-^/^-^ animals (112). In diabetic nephropathy models, GSK484 reduced NET formation, alleviated proteinuria and glomerular injury, and suppressed NET-driven NLRP3 inflammasome activation (113).

The therapeutic efficacy of PAD4 inhibition is not universal. In HFD-induced obese mice, neutrophil-specific PAD4 knockout (Ne-PAD4^-^/^-^) animals gained less weight over ten weeks of HFD feeding. They were also protected against obesity-induced cardiac remodeling and diastolic dysfunction. HFD did not, however, alter NET content in a venous thrombosis model. PAD4 therefore has organ-specific roles in obesity-related chronic inflammation (127). In experimental colitis, GSK484 reduced colonic mucosal NET density. Disease activity indices and inflammatory biomarkers did not improve. Optimal dosing remains to be established (128). Targeted delivery of GSK484 via collagen IV-directed nanoparticles achieved selective NET inhibition at sites of endothelial injury. Endothelial continuity was preserved. The strategy provides a methodological template for overcoming the poor specificity of systemic PAD4 inhibition (129). In STZ-diabetic rats, PAD4-targeted therapy reduced pancreatic NETosis markers and mitigated β-cell loss, suggesting a β-cell-protective role in type 1 diabetes. Translational relevance of this finding to obesity-driven T2DM has not been directly established. Caution is warranted when extrapolating across disease subtypes (130). No completed randomized controlled trial data are currently available for PAD4 inhibitors in obesity-related diabetes. Key challenges include disruption of innate immune defense with systemic administration and the limited oral bioavailability of existing formulations.

#### 6.2.2 Neutrophil elastase inhibitors

Neutrophil elastase (NE) acts in concert with myeloperoxidase (MPO) to degrade histones and promote chromatin decondensation. NE is a second key molecular mediator of NETosis. In obesity, NE activity is elevated and its endogenous inhibitor α1-antitrypsin is reduced. The combination contributes importantly to adipose tissue inflammation and insulin resistance (131). Sivelestat is a marketed selective NE inhibitor. It suppresses I*κ*B phosphorylation and NF-κB activation and thereby exerts anti-inflammatory effects (132). Note on disease-model translation: the following data on sivelestat and elafin were generated in non-obese diabetic (NOD) mice. NOD mice model autoimmune type 1 diabetes rather than obesity-associated T2DM. The data inform the conceptual role of NE in β-cell injury. They do not directly establish efficacy in T2DM. Early administration of sivelestat or its biological inhibitor elafin in NOD mice significantly reduced the incidence of insulitis and autoimmune β-cell destruction. The mechanism involves attenuation of macrophage infiltration and cytotoxic T-cell-mediated islet attack (84). Sivelestat effectively inhibits NE-induced NET formation *in vitro*. Systemic free-drug administration, however, showed no efficacy in a murine endotoxic shock model. Encapsulation in lipid-based nanoparticles substantially improved its *in vivo* NET-inhibitory efficacy and survival outcomes (133). NE inhibitors similarly attenuated proteinuria and glomerular injury in diabetic nephropathy models (113).

#### 6.2.3 Other strategies targeting NETosis

Reactive oxygen species (ROS) scavengers can block ROS-dependent NETosis. Examples include N-acetylcysteine and MitoTEMPO. Calcineurin inhibitors such as cyclosporine A suppress NETosis by disrupting calcium–calcineurin–NFAT signaling (134, 135). Metformin has glucose-lowering activity. It also inhibits the PKC–NADPH oxidase pathway and reduces circulating NET components in patients with T2DM and prediabetes. The NET components reduced include elastase, proteinase 3, histones, and cfDNA. Reduction is independent of glycemic control (125). Existing antidiabetic agents therefore harbor latent anti-NET therapeutic value. This point is developed in §6.6.3.

### 6.3 Therapeutic strategies targeting NET degradation

#### 6.3.1 DNase I therapy

DNase I degrades NETs by hydrolyzing their DNA backbone. Efficient *in vivo* NET clearance depends on coordinated activity of DNase I and DNase I-like protein 3 (DNase1L3). DNase I preferentially degrades double-stranded DNA. DNase1L3 preferentially degrades chromatin (136). In a rat model of IgA vasculitis, intravenous DNase I significantly reduced NET density in the kidney, gastric antrum, and duodenum. Renal MPO and CitH3 expression were also decreased. Target organ morphological injury was ameliorated (137). NETs promote resistance to fibrinolysis through PAD2/4-mediated fibrinogen citrullination. DNase I-mediated NET degradation reverses this antifibrinolytic effect. The strategy may help prevent thrombotic cardiovascular complications in diabetes (138). DNase I also degrades ATP and ADP to generate adenosine, exerting antithrombotic effects through a NET-independent pathway (139).

Inherent limitations constrain the clinical use of DNase I. The plasma half-life is short and stability *in vivo* is poor. NETs resist DNase I in infectious settings, where bacteria can stimulate platelets to release PF4 and interfere with DNase I activity. NET clearance is incomplete, and residual histones and MPO retain pro-inflammatory activity. Intravenous administration in septic shock causes dose-limiting hypotension (140). To address these limitations, investigators have engineered a recombinant dual-active DNase1 G,K,O variant. The variant combines DNase I and DNase1L3 activity. It demonstrates superior NET-degrading capacity *in vitro* compared with wild-type DNase I, though *in vivo* validation is pending (136). Clinical exploration of DNase I has begun in COVID-19-associated coagulopathy and ischemic stroke. Systematic evaluation in obesity-related diabetes remains absent.

### 6.4 Strategies blocking EV release or uptake

#### 6.4.1 Targeting EV biogenesis

EV biogenesis depends on inward budding of multivesicular body (MVB) membranes. Ceramide plays a critical regulatory role in this process. In obesity, ceramide and palmitate accumulation in adipocytes markedly promotes exosome production (141). GW4869 is a non-competitive inhibitor of neutral sphingomyelinase 2 (nSMase2). It reduces EV secretion by blocking ceramide synthesis. In ob/ob and HFD mouse models, GW4869 effectively blocked the transfer of miR-141-3p from obese adipose tissue exosomes to hepatocytes. The exosome-mediated hepatic insulin resistance pathway was disrupted (142, 143). In lean DIO mice, GW4869 administration (1 mg/kg/d for 16 days) mildly impaired glucose tolerance without affecting body weight or energy expenditure. This result confirms the physiological importance of adipocyte-derived EVs in glucose homeostasis. It also highlights why *selective* rather than blanket EV-blockade is required (50). Systemic EV biogenesis inhibition strategies lack cell-type specificity. They may inadvertently block protective EV signaling and generate unintended off-target effects (20).

#### 6.4.2 Blocking pathogenic EV uptake

EVs derived from obese adipose tissue carry miR-155, miR-34a, miR-29a, and others. Upon uptake by macrophages, these miRNAs promote M1 pro-inflammatory polarization. PPARγ expression is suppressed. Trans-organ propagation of insulin resistance follows (144). Selectively blocking pathogenic EV binding to recipient cells is one viable strategy under investigation. Another is neutralizing pathogenic miRNA cargo using antisense oligonucleotides. *In vivo*, large EVs engage in direct physical contact with NETs during systemic inflammation. Their interaction synergistically amplifies vascular inflammation and immunothrombosis (19). Disrupting this EV–NET physical interaction warrants deeper exploration in obesity-related metabolic inflammation.

### 6.5 Engineered EV-based drug delivery strategies

#### 6.5.1 Design principles and technical platforms

Engineered EVs (eEVs) employ the natural EV scaffold as a delivery framework. Therapeutic molecules are delivered through either exogenous cargo loading or genetic modification of parental cells. eEVs are characterized by low immunogenicity, high biocompatibility, and the capacity to traverse biological barriers. They are therefore a compelling platform for diabetes therapeutics (145). Loading strategies include co-incubation, electroporation, sonication, and endogenous loading via parental cell overexpression. The MISEV2023 guidelines provide a comprehensive framework for EV research standardization. They cover particle characterization, cargo identification, and quality control. The guidelines also offer a regulatory reference for clinical-grade eEV manufacturing (21). As of January 2025, 292 EV-related clinical trials had been registered on ClinicalTrials.gov; 170 were interventional. MSC-derived EVs accounted for 61% of therapeutic EV sources (146). Electroporation is the preferred method for high-efficiency insulin loading into exosomes. Insulin-loaded exosomes effectively promote glucose uptake in hepatocytes and fibroblasts *in vitro* (147).

#### 6.5.2 Adipose tissue macrophage-derived EVs

Adipose tissue macrophage (ATM)-derived small EVs (ATM-sEVs) are critical mediators of metabolic homeostasis in obesity-related diabetes. Their functional direction is dictated by the polarization and activation context of the parent macrophage rather than by a simple M1/M2 dichotomy. ATM-sEVs from lean mice improve insulin sensitivity. Those from obese mice drive insulin resistance. ATM polarization status therefore determines the metabolic direction of EV function (148). Of particular translational interest, in a rosiglitazone-treated obese mouse model of insulin sensitization, Rosi-ATM-sEVs (small EVs released by ATMs of rosiglitazone-treated obese mice) reversed obesity-induced glucose intolerance and insulin resistance *in vivo*. They did so without inducing the weight gain and hemodilution associated with rosiglitazone itself. The principal functional molecule was miR-690. It directly improved insulin sensitivity in adipocytes, myotubes, and both human and murine hepatocytes by suppressing *Nadk* (58). The published evidence pertains specifically to TZD-induced ATMs rather than to canonical IL-4-driven M2 macrophages. The term Rosi-ATM-sEVs is therefore preferred in this therapeutic context. Functional Rosi-ATM-sEVs or their specific miRNA cargo may constitute a novel therapeutic substrate for T2DM.

#### 6.5.3 MSC-derived EVs in obesity-related diabetes

MSC-derived EVs (MSC-EVs) contain abundant anti-inflammatory miRNAs, growth factors, and immunomodulatory proteins. They have broad therapeutic potential in obesity–diabetes models. In HFD-induced obese mice, exosomes from human Wharton’s jelly MSCs improved glucose tolerance. Hepatic steatosis and insulin sensitivity were ameliorated via FGF21–adiponectin axis activation. Thermogenic genes including UCP1 and Prdm16 were upregulated in adipose tissue. White adipose tissue browning was promoted through the AMPK–SIRT1–PGC-1α pathway (149). In an HFD/STZ-induced T2DM rat model, human umbilical cord MSC-derived exosomes restored phosphorylation of IRS-1 and Akt. GLUT4 membrane translocation was promoted. Hepatic glycogen storage increased. STZ-induced β-cell apoptosis was suppressed (150). Adipose tissue-derived exosomes from obese individuals exhibit upregulation of miR-155, miR-34a, and miR-29a. These miRNAs aggravate insulin resistance by suppressing PPARγ and IRS1 or driving macrophage M1 polarization (144). In HFD-fed mice, exosomes derived from long-term (8–12 weeks) HFD adipose tissue induced hepatocellular ER stress, inflammation, and insulin resistance. Exosomes from short-term (4-week) HFD exposure did not. Pathological reprogramming of adipose exosome cargo therefore depends on the duration of chronic obesity (151). APC-derived EVs play a pivotal role in midlife obesity. In middle-aged mice, APC-derived EVs lack miR-145-5p. L-selectin expression in ATMs is not suppressed. NF-κB-driven M1 inflammatory programming proceeds. Liposome-mediated targeted delivery of a miR-145-5p mimic prevented HFD-induced obesity in middle-aged mice (61).

#### 6.5.4 Engineered EVs targeting NETs

Combining engineered EV technology with anti-NET therapeutic agents is an emerging area of research. Collagen IV-targeted nanoparticle delivery of GSK484 achieved selective NET inhibition at sites of arterial intimal injury. Endothelial continuity was preserved (129). The strategy provides a methodological precedent for targeted delivery of PAD4 inhibitors to sites of pathological NET accumulation in adipose tissue or pancreatic islets. Sequential administration of DNase I- and NE inhibitor-loaded nanoparticle systems demonstrated effective NET clearance in an LPS-induced pulmonary fibrosis model. The systems also reduced circulating cfDNA, MPO, and NE levels in isolated neutrophils from COVID-19 patients. The proof of concept supports dual-target nanoparticle intervention in NET-driven inflammatory pathologies (152).

#### 6.5.5 Metabolic regulation by miRNA-engineered EVs

Macrophage-derived EVs enriched with miR-223 can be transferred from neutrophils to hepatocytes via LDLR-dependent transport. The transfer produces beneficial effects on non-alcoholic steatohepatitis (NASH) and hepatic insulin resistance *in vivo* (153). Exercise substantially increases EV output. The resulting EVs home preferentially to the liver. Their functional cargo ameliorates hepatic insulin resistance. The metabolic benefits of exercise-induced EVs may therefore be harnessed as a therapeutic intervention in obesity-related diabetes (154).

### 6.6 Current status of clinical trials and early evidence

#### 6.6.1 Overall clinical progress in EV therapy

A scoping review of 40 published human studies on EV-based interventions up to the end of 2023 concluded that the field remains exploratory: studies were small, largely uncontrolled, and highly heterogeneous in route and dosing, making firm conclusions on safety or efficacy premature (155). By January 2025, 170 interventional EV trials had been registered on ClinicalTrials.gov. The US FDA approval of the investigational new drug application for AB126, an unmodified neural cell-derived EV product, in January 2024 is an encouraging regulatory signal, but still an early one (146). Notably, most current studies rely on local delivery; systemic EV-based treatment for metabolic diseases such as T2DM remains uncommon (156).

#### 6.6.2 Clinical evidence for anti-NET strategies

Clinical support for targeting NETs is still indirect. Increased NET deposition has been observed in endomyocardial biopsies from patients with heart failure and diabetes, while PAD4 deficiency protects diabetic mice from cardiac and renal injury, providing a pathological and mechanistic rationale for anti-NET approaches (112). DNase I has entered early clinical testing in COVID-19-associated coagulopathy and ischemic stroke, but not specifically in obesity-related diabetes. PAD4 inhibitors are being developed mainly for autoimmune indications such as rheumatoid arthritis, and their path into metabolic disease remains undefined (125).

#### 6.6.3 Translational priorities: repurposing existing antidiabetic agents as anti-EV–NET therapeutics

At present, the most practical translational opportunity is not a first-in-class engineered EV product or a selective anti-NET agent, but mechanistic repurposing of established antidiabetic drugs that already show anti-NET or anti-NLRP3 activity. These agents have known safety profiles, established prescribing pathways, and realistic near-term clinical feasibility.

Metformin is the clearest example. Beyond its AMPK-linked metabolic effects, metformin suppresses the PKC-NADPH oxidase pathway and reduces circulating NET-associated components, including neutrophil elastase, proteinase 3, histones and cfDNA, in patients with T2DM or prediabetes, apparently at least partly independent of glycemic control (125). *In vitro*, it also attenuates PMA- and high glucose-induced NETosis at clinically relevant concentrations (125). These findings justify several straightforward next-step studies: prospective comparison of NET biomarkers in newly diagnosed patients receiving metformin versus alternative first-line therapy; mechanistic subgroup analyses within completed cardiovascular outcome trials; and perioperative assessment of NET persistence before and after bariatric surgery, especially in relation to trained immunity-like myeloid reprogramming (§3.6).

SGLT2 inhibitors are similarly attractive. By raising circulating beta-hydroxybutyrate, they can suppress NLRP3 inflammasome assembly and reduce macrophage IL-1beta production (123). This mechanism may contribute to the cardiovascular and renal benefits observed in major outcome trials. Because the EV-NET-NLRP3 feed-forward loop (§§5.3, 5.5) appears to depend on sustained NLRP3 activation, SGLT2 inhibitors may weaken a central amplification node without the immunosuppressive liabilities of direct NLRP3 blockade. Mechanistic substudies embedded in completed SGLT2 inhibitor trials could quantify effects on NET biomarkers, circulating EV miRNA signatures and, where feasible, adipose tissue biology.

Other licensed drugs also merit systematic biomarker evaluation. GLP-1 receptor agonists may influence the gut-adipose-NET axis by improving barrier function (§5.4). Pioglitazone is relevant because the biology of Rosi-ATM-sEVs was originally characterized in the rosiglitazone setting (58). Statins have well-established anti-inflammatory effects beyond LDL lowering, and colchicine is a clinically relevant NLRP3-modulating agent. Collectively, these drugs define a realistic “fast-translation” track that complements, rather than competes with, longer-horizon strategies such as engineered EVs or selective NET inhibitors.

That said, reductions in NET biomarkers should not be mistaken for proven clinical benefit. Whether biomarker modulation translates into fewer cardiovascular events, slower kidney disease progression, or lower mortality remains unknown. These links require pre-specified, hypothesis-driven prospective evaluation rather than retrospective data mining. The present framework is intended to prioritize the most testable candidates, not to overstate readiness for clinical use.

### 6.7 Limitations and perspectives

Current intervention strategies face four recurrent limitations. First, selectivity remains poor. DNase I, PAD4 inhibitors, and systemic inhibitors of EV biogenesis are not cell- or tissue-specific and may suppress protective NET functions in host defense as well as beneficial EV signaling, including exercise-associated metabolically protective EVs (20, 125). This problem is sharpened by the existence of insulin-sensitizing EV subpopulations, such as hepatocyte-derived exosomes enriched in miR-3075 (10) and Rosi-ATM-sEVs enriched in miR-690 (58). A non-selective EV blockade strategy could therefore remove both harmful and beneficial vesicle pools.

Second, pharmacokinetics are suboptimal. DNase I has a short plasma half-life; many PAD4 inhibitors require parenteral delivery; NETs may become resistant to DNase I degradation in infectious settings; and exogenously administered EVs are rapidly cleared by the reticuloendothelial system, limiting drug exposure at target sites (140, 145).

Third, EV manufacturing and standardization remain major obstacles. Batch-to-batch variability in EV production and purification is substantial, quality-control frameworks are still evolving, and GMP-compliant scale-up remains difficult. MISEV2023 provides an important regulatory and methodological scaffold, but not yet a full solution for clinical translation (21).

Fourth, clinical evidence remains weak. Rodent models do not recapitulate the polygenic architecture and long disease course of human obesity-related diabetes. In metabolic disease, clinical EV studies are still dominated by small, non-randomized exploratory trials, and no completed RCT dataset is currently available (155, 156). In addition, sustained EV-NET hyperactivity after metabolic improvement may reflect trained immunity at the bone marrow level (§3.6), implying that peripheral NET suppression alone may yield limited long-term benefit.

Two additional considerations deserve explicit incorporation into future study design. One is sex difference (§3.4.4): therapeutic windows, dosing, and biomarker thresholds may need sex-specific calibration, as sex-dependent variation in NETosis, adipose EV cargo and metabolic phenotype has already been observed in HFD-STZ models (81, 82). The second is gut-targeted delivery (§5.4). If the gut is a major trigger site of systemic NETosis, orally delivered, colon-targeted, or GLP-1RA-coupled local delivery of DNase I or PAD4 inhibitors may better preserve systemic innate immunity while protecting barrier function.

Taken together, the most credible next steps are combination strategies that pair selective NET inhibition with protective EV delivery, homing ligand-modified engineered EVs for adipose or islet targeting, stimulus-responsive “smart” EV systems, and prospective randomized trials in obesity-related T2DM incorporating NET biomarkers and EV cargo profiles as companion measures. Mechanistically, the EV-NET axis is compelling; evidentially, it remains largely preclinical. It should therefore be treated as a priority research agenda rather than a clinically deployable platform.

## 7 Research limitations and future perspectives

### 7.1 Standardization challenges in EV isolation and characterization

One of the central problems in EV research is the lack of broadly accepted standards for isolation and characterization. Common methods, including ultracentrifugation, size-exclusion chromatography and precipitation-based kits, differ substantially in particle recovery, purity and protein contamination (21). In plasma, EV concentrations are roughly 10¹¹ particles/mL, whereas lipoproteins may reach 10¹^6^ particles/mL; because their physical properties overlap, co-isolation is often unavoidable. Method-specific bias toward particular EV subpopulations further undermines cross-study comparability in proteomic and miRNA analyses (22).

MISEV2023 offers a structured framework for pre-analytical handling, method selection and reporting (22). Its evolution from MISEV2014 to the current third edition reflects real progress in standardization (3). Even so, several critical pre-analytical variables remain insufficiently resolved, including freeze-thaw cycles, storage buffer composition and biofluid-specific handling protocols (22). In obesity-related diabetes, a further common problem is failure to distinguish EVs derived from adipocytes, stromal vascular cells and adipose tissue macrophages, which obscures cell-specific contributions to insulin resistance and inflammatory signaling (141).

High batch variability in clinical-grade EV preparations and the immaturity of GMP-compatible production pipelines remain major translational bottlenecks (146). Circulating EV miRNA profiles as liquid biopsy biomarkers are also vulnerable to pre-analytical variability and contamination by blood cell-derived vesicles (157). Priorities therefore include cell-type-specific EV capture technologies, multicenter reference datasets, and clinically usable high-throughput single-particle analysis platforms (145).

### 7.2 Methodological limitations in *in vivo* NET detection

Reliable quantification of NETs *in vivo* remains difficult. Widely used approaches include MPO-DNA co-localization by immunofluorescence, plasma MPO-DNA complex ELISA and circulating CitH3 measurement (24). As discussed in §3.5, MPO-DNA ELISA is not a true gold standard, but rather the most widely used clinical surrogate, and the same caveat applies here. Most commercial anti-CitH3 antibodies show imperfect site specificity, and plasma calibrators are often unstable, contributing to poor reproducibility across studies (23). CitH3 is also not a universal NET marker, because some forms of NET formation occur independently of PAD4; reliance on CitH3 alone therefore risks systematic false negatives (126).

cfDNA and MPO-DNA complexes are frequently used as surrogate NET markers, but both lack specificity. cfDNA may derive from necrotic or apoptotic cells, and bacterial induction of platelet factor 4 can distort MPO-DNA quantification in infectious contexts (140). These sources of noise are especially problematic in chronic inflammatory states such as obesity-related diabetes, where it is difficult to disentangle NET dynamics from the broader burden of systemic inflammation (119). Real-time imaging of NETs *in vivo* is similarly constrained. Current methods rely largely on fluorescently labelled neutrophil microscopy, which is low-throughput and cannot distinguish suicidal, vital and mitochondrial NETosis (19). In adipose tissue, optical penetration limits and autofluorescence further hamper *in situ* imaging. More promising directions include multiparameter flow panels integrating CitH3, MPO-DNA and neutrophil elastase, as well as CyTOF-based high-dimensional phenotyping (24). Importantly, systematic studies linking NET biomarker levels to target-organ injury in obesity-related diabetes are still lacking; existing clinical data are mostly small, cross-sectional and analytically under-controlled (119).

### 7.3 Inherent limitations in translating animal models to human disease

HFD and HFD/STZ models reproduce insulin resistance and hyperglycemia within weeks, but differ fundamentally from human T2DM in genetic background, disease trajectory and sex-dependent phenotype. STZ-induced beta-cell toxicity compresses a long human disease course into a short experimental time window and therefore cannot model decades of progressive disease. Under similar conditions, male mice usually develop more marked insulin resistance than females, whereas sex effects in NETosis and EV biology add another layer of complexity (§3.4.4) (81). Species differences are also substantial: neutrophils comprise roughly 20% of circulating leukocytes in mice but about 50% in humans, and mouse neutrophils have a lower spontaneous tendency to form NETs. These differences may lead to systematic overestimation of anti-NET efficacy in animal models (158). Monogenic obesity models such as ob/ob and db/db likewise fail to capture the polygenic and environmental complexity of human obesity (159).

Disease subtype boundaries also matter. In this review, evidence derived from type 1 diabetes models has been explicitly labelled, including NOD mice (84), high-dose STZ models (130) and autoimmune insulitis paradigms. These findings should not be assumed to transfer directly to obesity-driven T2DM. Evidence from HFD, HFD/low-dose STZ, ob/ob, db/db and diet-induced obesity models is more relevant, but each still has clear limitations. For this reason, interventions supported only in T1DM settings, such as the sivelestat/elafin data in §6.2.2 and STZ-rat PAD4 data in §6.2.1, should continue to be interpreted cautiously.

Marked species differences in EV composition, abundance and surface proteomics further limit translational fidelity. Most rodent models also fail to reproduce the full spectrum of obesity-related diabetic complications, including retinopathy, nephropathy and cardiomyopathy, which are precisely the settings in which a systemic EV-NET axis may be most clinically relevant (19). Humanized models, patient-derived organoids, organ-on-chip systems and biopsy-based translational studies are therefore essential if this gap is to be narrowed (160).

### 7.4 Emerging applications of multi-omics, single-cell, and humanized-model technologies

Multi-omics and single-cell platforms are beginning to provide the resolution needed to dissect EV-NET interactions in obesity-related diabetes. scRNA-seq has already identified more than 60 cell populations in human white adipose tissue, revealing subpopulation-specific programs that are obscured in bulk analyses and linking certain cell states to insulin resistance and dyslipidemia (161). In neutrophil biology, integrated single-cell multi-omics has identified functionally distinct subpopulations with different NETosis tendencies in COVID-19, but comparable work in obesity-related diabetes is still limited (162).

Integration of scRNA-seq with spatial transcriptomics has also identified distinct ecological niches for lipid-associated macrophages in early obese adipose tissue (163). This suggests that EV-neutrophil communication is likely to be strongly microenvironment-dependent, a feature that conventional *in vitro* systems cannot capture. Future spatial studies should ideally examine EV cargo at near single-vesicle resolution, neutrophil infiltration and NET deposition within crown-like structures, and downstream signaling activation in target adipocytes within the same tissue context.

CITE-seq-based tracking of immune changes in obese mouse adipose tissue further suggests that obesity-induced immune imprinting can persist after weight loss, consistent with the trained immunity framework discussed in §3.6 and potentially with EV-mediated epigenetic information transfer (164). Single-cell multi-omics integrating transcriptomic, proteomic and epigenomic layers may therefore be particularly valuable for clarifying cellular heterogeneity and disease mechanism (165, 166).

Humanized models and organ-on-chip systems are equally important. Patient-derived adipose and islet organoids, as well as microphysiological systems, can better recreate human tissue structure, cellular composition and intercellular signaling than monolayer culture (160). Recent platforms include vascularized adipose chips for immune migration studies, islet chips for beta-cell function under fluctuating glucose, and multi-organ systems capable of probing the gut-adipose-NET axis (§5.4). For EV-NET research, these systems offer several advantages: quantitative dose-response analysis under physiologically relevant flow, longitudinal sampling without animal sacrifice, integration of patient-specific genetic and metabolic background, and a more tractable preclinical screen for candidate interventions.

Circulating EV miRNAs are also emerging as minimally invasive markers of metabolic state, with miR-26a, miR-126 and miR-223 among the more prominent candidates, all showing significant changes after intervention-driven weight loss (157). Longitudinal multi-omics profiling of adipose-derived EVs integrated with clinical metabolic data may ultimately support EV-NET biomarker panels for early detection and response monitoring in obesity-related diabetes (146, 167).

### 7.5 Key scientific questions awaiting resolution

Several questions now define the field. First, what initiates the EV-NET feed-forward loop, and in what temporal order does it emerge during progression from obesity to diabetes? It remains unclear whether adipocyte-derived EVs directly trigger neutrophil activation and NET formation, whether NET-associated inflammation first amplifies EV release, or whether the dominant direction changes across disease stages. Answering this will require combined genetic models of adipocyte-specific EV secretion defects and neutrophil-specific PAD4 deletion, alongside prospective human cohorts (19, 112).

Second, the relative contribution of the EV-NET axis across organs remains poorly defined. Independent evidence links this pathway to beta-cell injury, adipose inflammation, NASH progression and diabetic kidney disease (112, 115), but how these organ-specific effects are coordinated through circulating EVs is still unclear.

Third, the dynamic balance between protective and pathogenic EV populations remains unresolved. Exercise-induced EVs and miR-690-rich Rosi-ATM-sEVs appear metabolically beneficial, whereas obese adipose-derived EVs promote insulin resistance (58). Hepatocyte exosomes enriched in miR-3075 in early-onset obesity models further suggest that protective EV subpopulations may have been underestimated (10). Whether this balance can be pharmacologically shifted toward protection remains unknown.

Fourth, existing anti-NET strategies may interfere with beneficial EV signaling. Systemic PAD4 inhibition could alter neutrophil-derived EV secretion or function, and GW4869 might suppress protective as well as pathogenic EV production (50, 143). These issues are central to rational design of selective interventions.

Fifth, a human tissue-based EV-NET research framework is still needed. The field will require coordinated patient cohorts spanning the full spectrum of obesity severity and diabetes duration, paired with patient-derived organoids and microfluidic humanized systems (160).

Sixth, the contribution of trained immunity to persistent NET hyperactivity after metabolic improvement remains uncertain. If bone marrow-level immune reprogramming sustains NET production after bariatric surgery, drug therapy or lifestyle intervention, targeting myeloid progenitor epigenetic states may prove more durable than peripheral NET suppression alone. Longitudinal human studies with paired bone marrow and peripheral blood sampling before and after intervention will be especially informative.

Finally, sex-stratified EV-NET biology requires direct study. Existing data indicate sex-dependent differences in both NETosis and EV cargo (§3.4.4), raising the possibility that biomarker thresholds and therapeutic windows differ between male and female patients. Stratification around the menopausal transition is particularly relevant, because estrogen can suppress PAD4 expression and neutrophil NETosis, potentially altering both disease risk and treatment response (82).

## 8 Conclusion

### 8.1 EVs and NETs constitute a sterile autoinflammatory circuit in obesity-induced T2DM

The central conclusion of this review is that EVs and NETs may form a self-reinforcing sterile inflammatory circuit in obesity-driven T2DM. Chronic low-grade adipose inflammation in obesity promotes EV release from adipocytes and immune cells; these EVs carry pro-inflammatory cargoes such as miR-155 and miR-34a that can activate local neutrophils and promote NETosis; in turn, NET-derived cfDNA, histones, neutrophil elastase and myeloperoxidase may stimulate surrounding cells to release additional EVs, thereby amplifying the loop (19).This model is appealing because it offers a mechanism by which local adipose inflammation might be translated into systemic, multi-organ injury affecting pancreatic beta cells, hepatocytes, glomerular cells and cardiomyocytes through circulating EV-mediated signaling (111, 131). At the same time, not all obesity-associated EVs are pathogenic. Hepatocyte-derived exosomes enriched in miR-3075 (10) and miR-690-rich Rosi-ATM-sEVs (58) appear insulin-sensitizing rather than harmful. Any clinically credible intervention must therefore distinguish pathogenic from protective EV pools rather than treating all EVs as equivalent targets.

Within this framework, obesity-induced insulin resistance increases adipocyte stress and promotes release of pathogenic EVs, whereas persistent NET formation aggravates beta-cell dysfunction and tissue insulin resistance, further sustaining the cycle (131, 148). The model integrates previously separate EV and NET literatures and identifies several pharmacologically tractable nodes, including ceramide/nSMase2-dependent EV biogenesis, PAD4- and neutrophil elastase-dependent NET formation, and the EV-NET interaction interface. However, many of the core interactions discussed in §§4 and 5, especially the EV to NET to EV amplification loop, are still inferred in part from sepsis, lupus and severe COVID-19 rather than directly demonstrated in human obesity-related T2DM. The axis should therefore still be regarded as a high-priority mechanistic hypothesis, not an established clinical fact (§4.5 and §7.5).

### 8.2 Therapeutic potential of targeting the EV–NET axis: promise and present limitations

Targeting the EV-NET axis is mechanistically attractive, but the supporting evidence remains weighted toward preclinical work. At present, the evidence can be viewed in three tiers.

The first tier is preclinical rodent evidence. PAD4 inhibition with GSK484 has shown protective effects in diabetic models of retinopathy and cardiorenal injury (127). Neutrophil elastase inhibition reduces insulitis in NOD mice (84). DNase I-mediated NET clearance promotes regression of atherosclerosis in diabetic mice (17)., and PAD4 deficiency preserves beta-cell mass (112, 168). In parallel, Rosi-ATM-sEVs carrying miR-690 improve insulin sensitivity in obese mice without the weight gain or hemodilution associated with thiazolidinediones (58). These are important proof-of-concept data, but disease subtype matters: NOD is a model of autoimmune T1DM, not obesity-driven T2DM, and extrapolation across subtypes should remain cautious.

The second tier is limited but genuine human evidence in metabolic disease. Metformin reduces circulating NET-associated components in patients with T2DM or prediabetes, apparently at least partly independently of glucose lowering (125). SGLT2 inhibitors suppress NLRP3 inflammasome activity through elevation of beta-hydroxybutyrate, a mechanism that may help explain part of their cardiovascular and renal benefit (123). For now, these two drug classes represent the most realistic clinical entry points for EV-NET modulation and should be prioritized in prospective studies (§6.6.3).

The third tier consists of conceptually appealing but unvalidated strategies. Engineered EVs carrying adipose- or islet-targeting ligands could in principle deliver PAD4 inhibitors or DNase I directly to sites of pathological NET accumulation (129). Likewise, circulating NET biomarkers and adipose-derived EV miRNA signatures might be combined into companion diagnostics for patient stratification and response monitoring (157, 167). Yet no completed randomized trial in obesity-related T2DM has validated either approach, and manufacturing, quality control and regulatory frameworks for engineered EV therapeutics remain immature (146, 155, 156).

### 8.3 A Call for multidisciplinary collaboration to bridge the bench-to-bedside gap

The EV-NET axis remains, first and foremost, a mechanistic proposition. It is supported by animal work and selected indirect human data, but not yet by adequately powered interventional trials. Moving this field toward clinical application will require progress across materials science, manufacturing, disease modelling, clinical investigation and data integration.

Materials science and bioengineering must improve standardized EV isolation and targeted delivery; nanomedicine and pharmaceutics must address GMP production and *in vivo* stability of engineered EVs and anti-NET agents (146); and translational research must develop models that more faithfully capture the complexity of human obesity-related diabetes, including humanized animals, patient-derived organoids and organ-on-chip platforms (159, 160). Clinical progress will also depend on coordinated work across endocrinology, nephrology, cardiology, ophthalmology and immunology to build patient cohorts with meaningful multi-organ endpoints. Computational biology and bioinformatics will be equally important for integrating increasingly complex multi-omics datasets into testable mechanistic models rather than descriptive inventories (165, 166).

Several design challenges should be addressed upfront in future clinical studies. Existing EV trials in humans remain small and largely uncontrolled, with no completed randomized datasets in metabolic disease (155, 156). More fundamentally, the therapeutic window must suppress pathogenic NETosis without impairing beneficial innate immune function or insulin-sensitizing EV populations (138). Sex stratification (§3.4.4), persistence of trained immunity-driven NET hyperactivity after metabolic improvement (§3.6), and the possibility that the gut is a principal trigger site (§5.4) should all be treated as prospective design variables rather than retrospective explanations.

### 8.4 Actionable clinical takeaways

Although current evidence is insufficient for direct clinical guidance, it is sufficient to support several low-friction translational priorities.

First, combination biomarker panels should be tested for risk stratification and treatment monitoring. A rational panel would include plasma MPO-DNA complexes, CitH3, cfDNA and selected circulating EV-miRNA features such as miR-155, miR-27a-5p, miR-130b and miR-3075. Potential uses include identifying individuals with obesity or prediabetes at high risk of progression to T2DM, stratifying patients at elevated risk of retinopathy, nephropathy or cardiomyopathy, and monitoring responses to anti-inflammatory or anti-NET interventions. Because no single marker is sufficient (§3.5, §7.2), at least two orthogonal markers should be incorporated whenever possible.

Second, metformin and SGLT2 inhibitors should be treated as the most realistic near-term candidates for mechanistic repurposing. In both new prospective cohorts and randomized substudies within existing cardiovascular outcome trials, NET and EV biomarkers should be embedded as key secondary endpoints to determine how much modulation of the EV-NET axis contributes to observed clinical benefit.

Third, combination strategies that target both arms of the loop deserve careful testing. Examples include background therapy with metformin or an SGLT2 inhibitor combined with selective PAD4 inhibition or targeted DNase I delivery; pairing systemic treatment with organ-targeted delivery platforms, such as type IV collagen-targeted GSK484 nanoparticles for vascular complications (129) or locally restricted intestinal DNase I/PAD4 inhibition in settings relevant to the gut-retina axis (111); or combining neutralization of pathogenic EVs with delivery of protective EV populations such as miR-690-rich Rosi-ATM-sEVs or miR-3075-rich hepatocyte EVs (10, 58). Timing will matter and should be defined empirically.

Fourth, early clinical studies should focus on patient subgroups most likely to yield interpretable biological signals: newly diagnosed T2DM with elevated baseline NET biomarkers; patients who show metabolic improvement after bariatric surgery but retain high NET activity, consistent with trained immunity-like persistence; and patients with early microvascular complications such as retinopathy or microalbuminuria, in whom the gut-adipose-NET axis (§5.4) may offer a tractable intervention point. All such studies should incorporate sex stratification and report sex-specific outcomes.

In summary, the sterile autoinflammatory positive feedback circuit constituted by the mutual promotion of EVs and NETs is a mechanistically compelling framework for understanding obesity-related T2DM and its multi-organ complications. The framework identifies clear molecular targets and clear translational opportunities. The field is, however, at an early stage. Realizing the framework’s clinical promise will require coordinated advances in multidisciplinary collaboration, methodological standardization, and—above all—adequately powered prospective human studies. We hope this review will help orient those efforts.
